# Structural insights into the promiscuous DNA binding and broad substrate selectivity of fowlpox virus resolvase

**DOI:** 10.1038/s41598-019-56825-w

**Published:** 2020-01-15

**Authors:** Na Li, Ke Shi, Timsi Rao, Surajit Banerjee, Hideki Aihara

**Affiliations:** 1 Department of Biochemistry, Molecular Biology, and Biophysics, University of Minnesota, 6-155 Jackson Hall, 321 Church Street S.E. Minneapolis, Minneapolis, MN 55455 USA; 2 Northeastern Collaborative Access Team, Cornell University, Advanced Photon Source, Lemont, Illinois 60439 USA; 3 Present Address: College of Biological Engineering, Henan University of Technology, Zhengzhou, 450001 People’s Republic of China

**Keywords:** DNA, X-ray crystallography

## Abstract

Fowlpox virus resolvase (Fpr) is an endonuclease that cleaves a broad range of branched DNA structures, including the Holliday junction (HJ), with little sequence-specificity. To better understand the mechanisms underlying its relaxed substrate specificity, we determined the crystal structures of Fpr and that in a novel complex with HJ at 3.1-Å resolution. In the Fpr-HJ complex, two Fpr dimers use several distinct regions to interact with different DNA structural motifs, showing versatility in DNA-binding. Biochemical and solution NMR data support the existence of non-canonical modes of HJ interaction in solution. The binding of Fpr to various DNA motifs are mediated by its flat DNA-binding surface, which is centered on a short loop spanning K61 to I72 and flanked by longer α-helices at the outer edges, and basic side grooves near the dimer interface. Replacing the Fpr loop K61~I72 with a longer loop from *Thermus thermophilus* RuvC (E71~A87) endows Fpr with an enhanced selectivity toward HJ cleavage but with a target sequence preference distinct from that of RuvC, highlighting a unique role of this loop region in Fpr-HJ interaction. Our work helps explain the broad substrate selectivity of Fpr and suggests a possible mode of its association with poxvirus hairpin telomeres.

## Introduction

Telomeres protect the ends of a linear chromosome and facilitate complete replication of terminal DNA sequences. One of the simplest forms of telomere is a covalently closed hairpin structure, which is found in bacteria and viruses carrying linear chromosomes. Replication of a poxvirus linear chromosome entails single nicking near one of the hairpin termini followed by a unidirectional strand displacement synthesis, dubbed as “rolling-hairpin” replication^1^. This replication mode would generate a linear head-to-head, tail-to-tail array of chromosomes that needs to be resolved at the inverted repeat junctions. It is believed that the inverted repeat junctions in the poxvirus replication intermediate form a cruciform structure with extruded hairpins, which are processed by a virally encoded Holliday junction (HJ) resolvase^2–5^. The Holliday junction (HJ), a four-way double-stranded branched DNA structure originally proposed by Robin Holliday^6^ as an intermediate of genetic recombination, plays key roles in many biological processes. Various types of branch-specific endonucleases capable of cleaving DNA strands in a HJ have been reported from both prokaryotes and eukaryotes.

The enzyme responsible for resolving the concatenated replication intermediate into unit length genomes in *Poxviridae* family was first identified and characterized in vaccinia virus (VV)^7^. The resolvase enzymes from fowlpox virus that causes disease in poultry and a closely related canarypox virus, have been studied biochemically and structurally^8–11^. The poxvirus resolvases belong to the RNaseH/retroviral integrase superfamily of DNA-modifying enzymes that employ a conserved metal-dependent mechanism^7,8,12,13^, including the prototypical bacterial RuvC HJ DNA resolvase. However, unlike bacterial RuvC that cleaves HJ at the (A/T) TT↓(G/C) cognate sequences^8,9^ across the junction point, the poxvirus resolvases do not exhibit sequence specificity in HJ resolution. Moreover, poxvirus resolvase can bind to a wide variety of DNA structural motifs, including HJ, bulged loops, three-way (Y) junction, splayed duplex, three stranded Holliday-like junction^8,9^. The broad substrate specificity has allowed development of a high-throughput screen to identify poxvirus resolvases inhibitors^8–10^. Even though the RuvC-HJ complex structure was elucidated by X-ray crystallography^14^ and a canarypox virus resolvase crystal structure has also been reported recently confirming the structural similarity between the two proteins, it remains unknown how poxvirus resolvase interacts with a variety of DNA substrates and the mechanism of its broad cleavage activity is still not fully addressed^11^.

In this study, we determined the crystal structures of the resolvase from fowlpox virus and its complex with a HJ DNA containing two hairpins. The fowlpox virus resolvase (Fpr) forms a stable dimer as other homologous resolvases^14–17^ and was found to bind to various structural motifs on the HJ DNA, revealing flexibility of the Fpr dimer in binding to a wide range of DNA structures. Solution studies confirm that Fpr and HJ DNA form two kinds of complexes with different Fpr: HJ ratios, and they are in dynamic exchange. Structural comparison with RuvC suggests that the loop region of Fpr spanning K61~I72 in the middle of the DNA-binding surface is a major determinant for the sequence-independent HJ DNA resolution. Replacing the loop region of K61~I72 with *Thermus thermophilus* RuvC E71~A87 endows Fpr with sequence-dependent cleavage activity, generating a resolvase (CRFpr) with novel sequence selectivity in HJ resolution.

## Results

### Structure of the Fpr dimer

The full-length 156-residue Fpr has been refractory to crystallization^11^. We found that the full-length Fpr is highly prone to oxidation and forming an intramolecular disulfide bond, causing sample aggregation and promoting heterogeneity. Removal of the C-terminal 6 amino acid residues (^151^CKISKD^156^) successfully prevents formation of the disulfide bond and has no appreciable effect on the catalytic activity. Thus we used a 150-residue Fpr (C151stop) in subsequent studies. Initial crystallization of C151stop Fpr with a catalytic mutation D135N to abolish the nuclease activity, in the presence of a HJ DNA yielded large crystals that diffracted only up to 7~8 Å resolution. Four additional surface conformational entropy reduction mutations, K65A, K102A, K103A, and Q104A, helped improve crystal quality and the structure was determined and refined to 3.1 Å. A HJ DNA substrate, HJ 13/13 with four 13 bp arms (Supplementary Table S1), was required for crystallization of Fpr and present in the washed crystals. However, no DNA molecules were observed in the electron density map and the structure obtained was that of Fpr in the APO (DNA-free) form.

**Figure 1:**
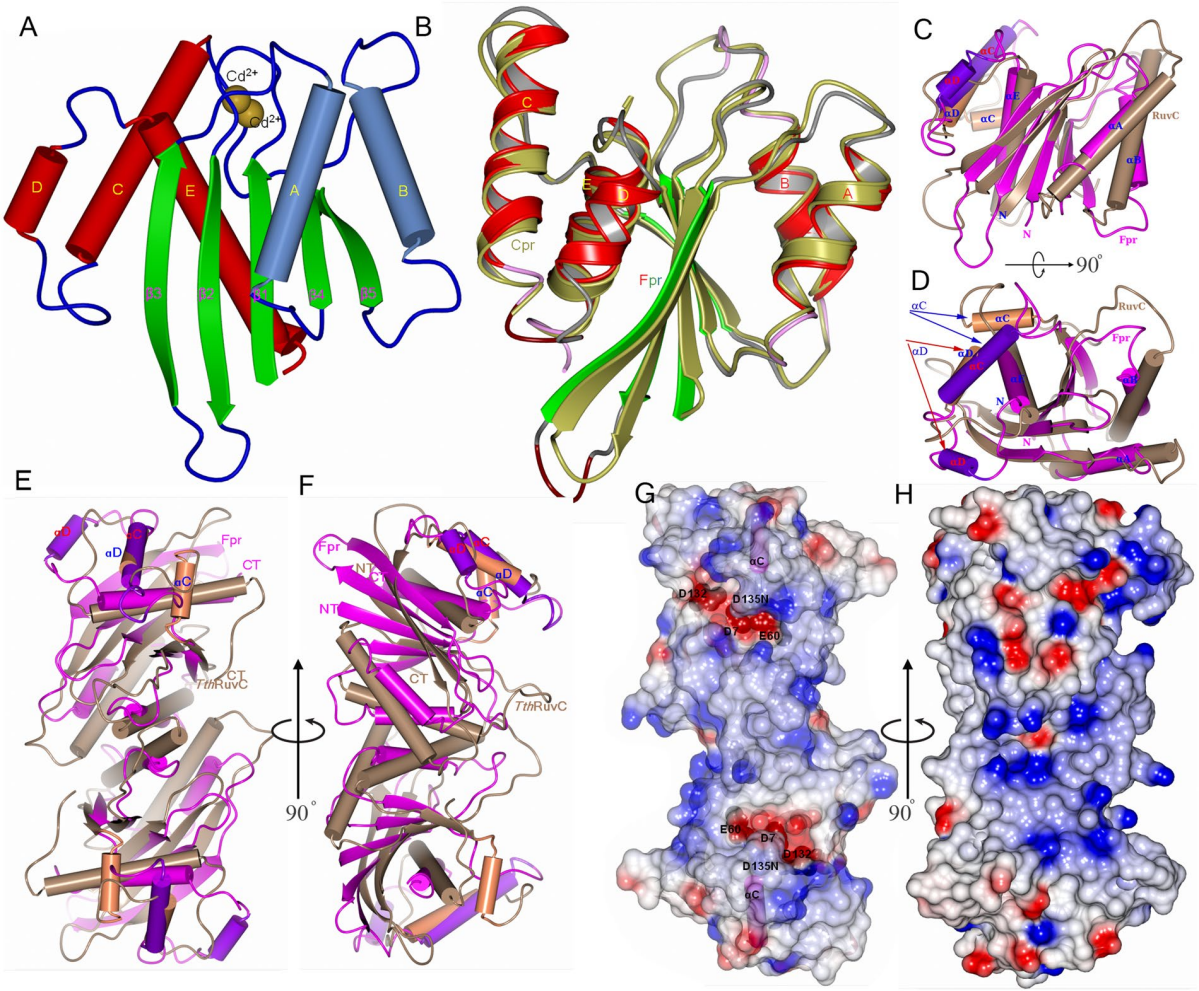
Overall structure of the fowl poxvirus resolvase (Fpr). (**A**) Structure of Fpr with the secondary structure elements labeled. (**B**) Superposition of Fpr and Cpr monomers based on the central five β-strands. (**C**) Superposition of Fpr and RuvC from T. *thermophiles* (*Tth*RuvC) monomers, highlighting that two proteins have different αC and αD helices (colored differently from the rest of the monomers and indicated by arrows). αB and the preceding loop in *Tth*RuvC are longer than the counterparts in Fpr, while αC in Fpr superposes onto αD of *Tth*RuvC but is much longer. (**D**) 90° rotation of C around a horizontal axis. (**E**,**F**) Superposition of Fpr and RuvC dimers showing the different shape of Fpr and *Tth*RuvC, F is 90° rotation of E around a vertical axis. (**F**) Electrostatic surface representation of the Fpr dimer. The negative surface (in red) formed by the active site residues can be seen at the bottom of the DNA binding grooves. The αC helices are shown in magenta. (**H**) 90° rotation of G around a vertical axis, showing strong positive potentials (blue) on the side of active site of the Fpr dimer. The orientations of G and H are same as E and F respectively.

The 150-residue Fpr protein has the characteristic architecture of enzymes from the retrovirus integrase/RNase H superfamily, comprising a single layer of β-sheet sandwiched between α-helices^18^. A five-pleated β-sheet forms the conserved core, which is flanked by two α-helices (αA and αB) on one side and three α-helices (αC, αD, and αE) on the other (Fig. 1A and B). A cluster of acidic side chains located at one end of the β-sheet form the active site of the enzyme, the DDE motif. Divalent cadmium ion used in the crystallization reservoir solution at 10 mM concentration, was identified in the active center pocket, coordinated by the acidic active site residues, D7, E60, D132, and N135 (D135 in the wild-type). The coordinating water molecules of the Cd^2+^ ions were not modeled due to limited resolution (supplementary Figure S1A). Two Fpr protomers form a homodimer, similarly to the homologous canary poxvirus resolvase (Cpr, PDB ID: 5E6F)^11^ and bacterial RuvC (PDB ID: 4EP4, 4LD0, 4KTW)^14,16,17^. The symmetrical homodimeric architecture is a general feature shared by various types of HJ-resolving enzymes^19–23^. In the Fpr dimer, αB helices from each protomer form the major dimer interface. The dimer interface is mainly formed by the hydrophobic interactions, including F64, Y73, F74, F78, and Y80 (Supplementary Figure S1B). Superposition of Fpr and canary poxvirus resolvase shows their similar monomer folding (Fig. 1B) and similar dimer configuration. A notable difference is that the Cpr crystal structure does not have clear electron density for the helix αD, whereas the Fpr structure shows a well ordered αD helix with clear electron density. Bacterial RuvC (Fig. 1C,D) does not have the corresponding helix, but it is present in yeast mitochondrial resolvase Ydc2^24^.

It has been found that *Thermus thermophilus* RuvC (*Tth*RuvC) binds specifically to an unstacked Holliday junction and cuts selective DNA sequence, while poxvirus HJ resolvase does not have strong sequence specificity and can bind to a variety of DNA structural motifs, including unstacked HJ-DNA, Y-shaped DNA, ssDNA, buldges and loops^8–10,14,16^. The superpositions of Fpr and *Tth*RuvC monomer are shown in Fig. 1C and D. The αB helix and the preceding loop at the dimer interface in *Tth*RuvC is much longer than those in Cpr and Fpr, which spatially separates the two metal-bound active site from each monomer. In contrast, the active sites and the surrounding DNA-binding surfaces of the two Fpr monomers are not well separated due to the shorter αB and the loop preceding the αB helix, and they form a larger flat pocket (Fig. 1E,F). The dimer interface of Fpr is 682 Å^2^ as compared to 1210 Å^2^ in *Tth*RuvC. αC and αD are positioned at the distal end of each monomer of the Fpr dimer. αC of Fpr structurally corresponds to, but is longer than, the fourth helix (αD) of *Tth*RuvC. The longer αC of Fpr form pronounced edges at the outer peripheries of the protein dimer surface that would likely make direct DNA contacts, as suggested for Cpr^11^. The residues mutated to improve crystal quality (K102/K103/Q104) are located at the N-terminal end of this helix. These differences lead to the unique DNA strand binding groove of Fpr. Both the top (Fig. 1G: harboring the active site residues D7, E60, D132 and D135N) and side (Fig. 1H) views of the surface of the Fpr dimer show clusters of positive electrostatic potential.

### Effect of surface mutations on DNA-binding and HJ resolution

**Figure 2:**
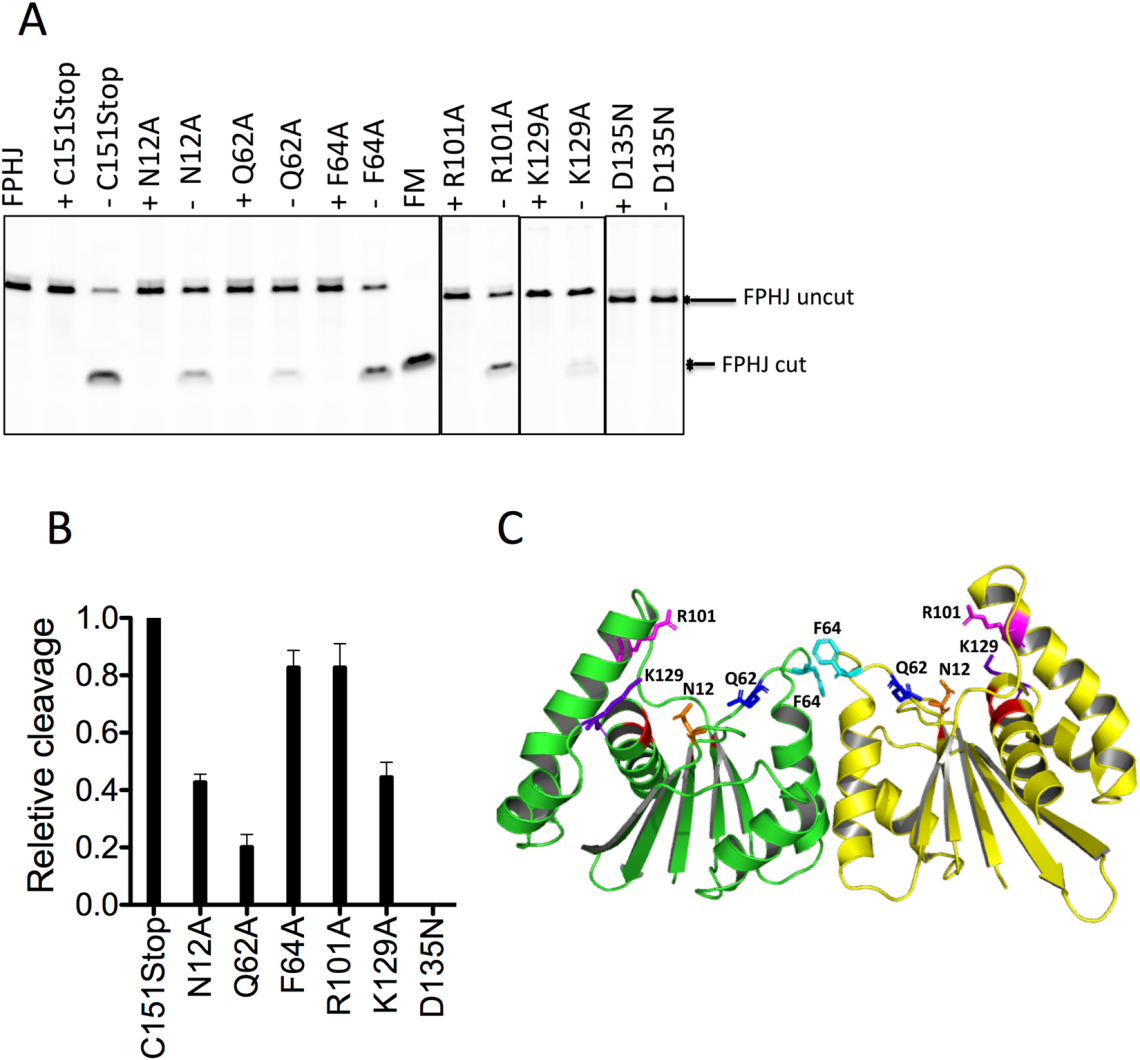
HJ resolution activity of select Fpr C151Stop mutants. (**A**) Cleavage of fluorescently labeled FprHJ DNA by Fpr with amino acid substitutions for residues near the active site, monitored on denaturing gels. The reaction mixture (20 μl) containing Fpr (300 nM) with FprHJ (100 nM) was incubated at 37 °C for 10 min. FprHJ, FprHJ only in reaction buffer as a control. FM, FprHJ marker (nicked duplex product). For each protein a sample containing 5 mM EDTA (+) served as the negative control, and that containing 5 mM MgCl_2_ without EDTA (−) shows the enzymatic activity. Borders of cropped gels are highlighted by black lines. (**B**) Quantitation of the cleavage products of above mutants relative to that of Fpr C151Stop. (**C**) Structure of the Fpr dimer with side chains of the mutated residues shown. Backbone of the catalytic residues are colored in red. Supplementary Figure S2 shows activities of the other mutants tested. Each DNA cleavage experiment was repeated 3 times and a representative cropped gel is shown in the figure. Original full-length gels for Fig. 2 are shown in Supplementary Figure S10.

Based on the structure of Apo Fpr dimer, 24 surface residues (Supplementary Figure S2A) were mutated to alanine, and the HJ-DNA cleavage activities of those mutations were examined. Fpr C151Stop with N12A, Q62A or K129A mutation showed greatly reduced DNA cleavage activity (Fig. 2A and B). Fpr C151Stop with F64A or R101A mutation showed a more modest decrease in cleavage activity, and other surface residue mutations did not significantly affect the cleavage activity (Supplementary Figure S2C–E). The Q62A mutant with reduced cleavage activity still resolved HJ by cleaving the same position as Fpr C151Stop (Supplementary Figure S2B). N12, Q62, R101, and K129 surround the active site of Fpr and F64 is near the dimer interface (Fig. 2C). These results confirm that engagement of DNA strand by the residues in the basic groove surrounding the active site is important for HJ resolution by Fpr.

**Figure 3:**
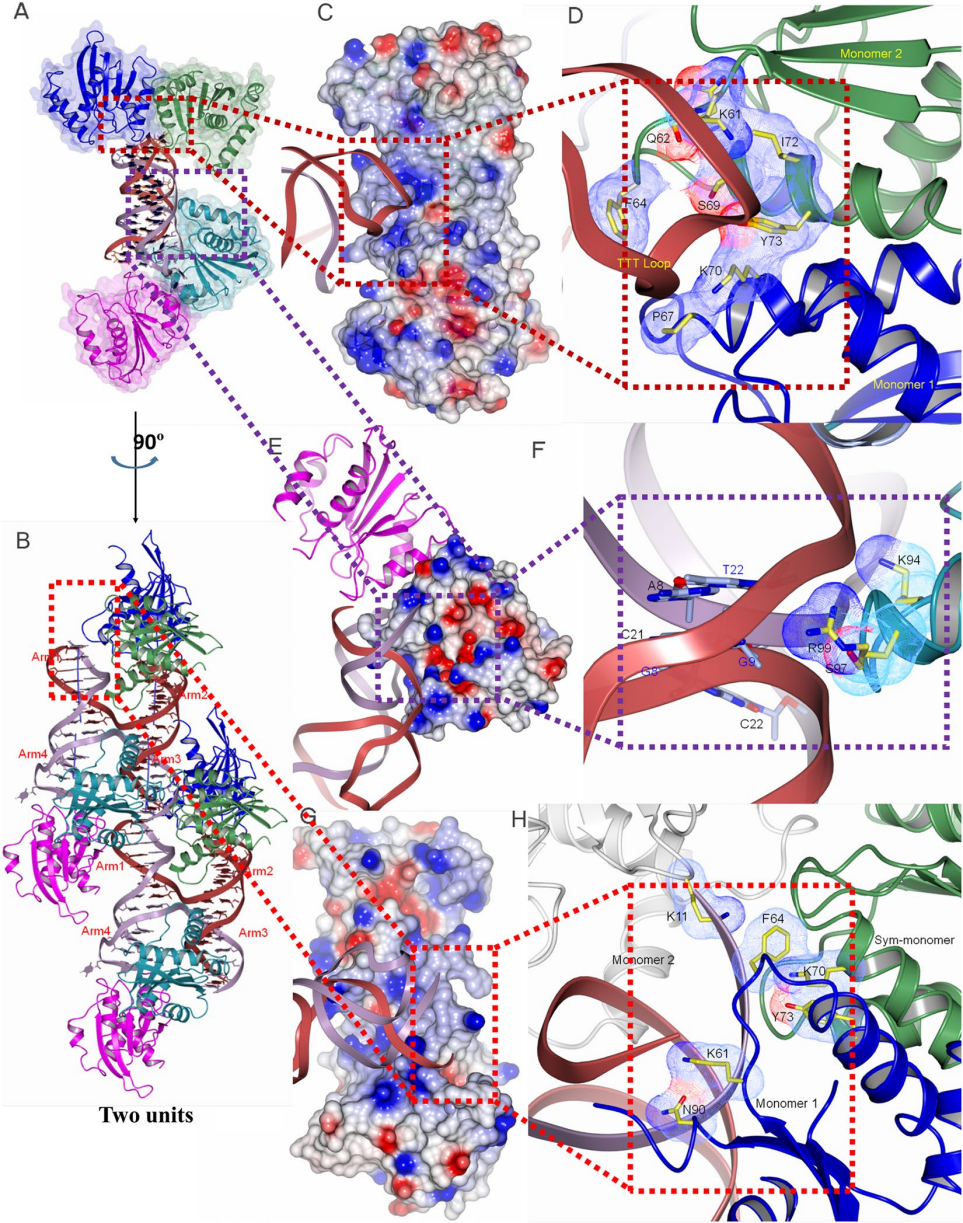
Crystal packing and several distinct DNA-binding modes observed in the Fpr-HJ8/5 complex. (**A**) Overall structure of the Fpr-HJ8/5 complex with two Fpr dimers bound to one HJ. (**B**) A view after 90º rotation around a vertical axis, showing two copies of the crystallographically independent unit. (**C**) The -TTT- loop bound in the basic groove formed at the side of the dimer interface. (**D**) The amino acids close to the bound -TTT- loop shown in sticks with their van der Waals radii highlighted by fine dots. (**E**) Contact between a Fpr molecule and HJ8/5 DNA at the cross-over junction point. (**F**) The amino acids interacting with HJ DNA near the junction point. (**G**) Interaction of a DNA blunt end with Fpr near the dimer interface. (**H**) The amino acids in the vicinity of the bound DNA double strand.

Binding affinities of these mutants to the 13/13 HJ DNA were also examined by fluorescence polarization assay (Supplementary Table S3). K11A, K34A and K70A mutations slightly reduced the binding affinity of Fpr C151Stop, indicating that these residues are involved in HJ binding. All of them are positively charged residues that interact with DNA in the Fpr-DNA complex described below (Fig. 3B,D). Notably, Fpr C151Stop N68A and R101A showed higher affinity than Fpr C151Stop. It may be caused by a conformational perturbation in Fpr to help accommodate HJ. Other mutations did not affect the binding significantly. Overall, the effects of single amino acid substitutions on HJ binding affinity were subtle, likely reflecting that Fpr has a broad positively charged surface involving many amino acid residues that can serve to interact with DNA.

### Fpr–HJ complex structure reveals a novel ‘sandwich’ binding mode

A variety of HJ DNA substrates were tested in an attempt to obtain the Fpr/HJ-DNA complex crystal structure. Eventually, the Holliday junction HJ8/5, which has two 5 bp arms covalently closed with -TTT- hairpins and 8 bp arms with blunt ends (Supplementary Figure S3A), gave crystals that diffracted to ~3.3 Å resolution. Crystal structure was solved by the SAD phasing method using a mercury derivative, with a FOM of 0.34. The electron density map shows clearly both Fpr and HJ-DNA in the crystal lattice. Unexpectedly, the crystal structure of the Fpr-HJ8/5 complex reveals that the HJ is in a H-stacked conformation, where arms 1 and 4 (as well as arms 2 and 3) of HJ8/5 stack on each other to form a pseudo-continuous double-helix. The two pseudo-continuous helices are linked by a crossover of backbones and are arranged in an antiparallel orientation with an angle between their axes of ~30° (Supplementary Figure S3B).

In the Fpr-HJ8/5 complex crystal lattice, an asymmetric unit contains one DNA Holliday junction and two copies of Fpr dimers (4:1 Fpr monomer: HJ ratio, Fig. 3A). The blunt ended DNA helices of Arms 1 and 3 also stack onto symmetry-related DNA molecule in the crystal, further extending the pseudo-continuous helices (Fig. 3B). The electron densities clearly show the interface formed by the stacking between the symmetry-related DNA molecules. Each HJ is surrounded by a total of 4 Fpr dimers (including two symmetry-related dimers) (Fig. 3B and Supplementary Figure S4). The Fpr dimers make extensive contacts and bridges between HJ-DNA molecules. These interactions are formed with various structural motifs from different regions of the HJ-DNA (Fig. 3C–H): TTT hairpin-loop, the crossover junction, and double-stranded DNA. The -TTT- hairpin-loop fits into a positively charged groove at the dimer interface formed between αA/αB and αB/β5 of the two monomers (Fig. 3C,D and Supplementary Figure S4). This interaction includes DNA backbone contacts by Trp41, Pro67, Lys70, and Tyr73, as well as electrostatic and van der Waals contacts with a flipped-out thymine base by Phe64, Ser69, and Ile72. At the center of HJ-DNA, the N-terminus of the αC helix and the preceding loop (between β5 and αC) contact the crossover junction, with a stretch of residues Ser97-Tyr98-Arg99 at the apex positioned over the DNA minor groove (Fig. 3E,F and Supplementary Figure S4). DNA strands near the stacked blunt ends are bound in the basic groove across the Fpr dimer interface, similarly to the -TTT- hairpin-loop but on the opposite side of the Fpr dimer. This interaction is centered around Phe64/Lys70/Tyr73, with flanking residues Lys61 and Asn90 positioned over the DNA minor groove. The symmetrically positioned basic side grooves are a unique feature of pox viral resolvases, formed due to their shorter αA compared to the corresponding αA helix of bacterial RuvC (Fig. 1 and Supplementary Figure S1).

**Figure 4:**
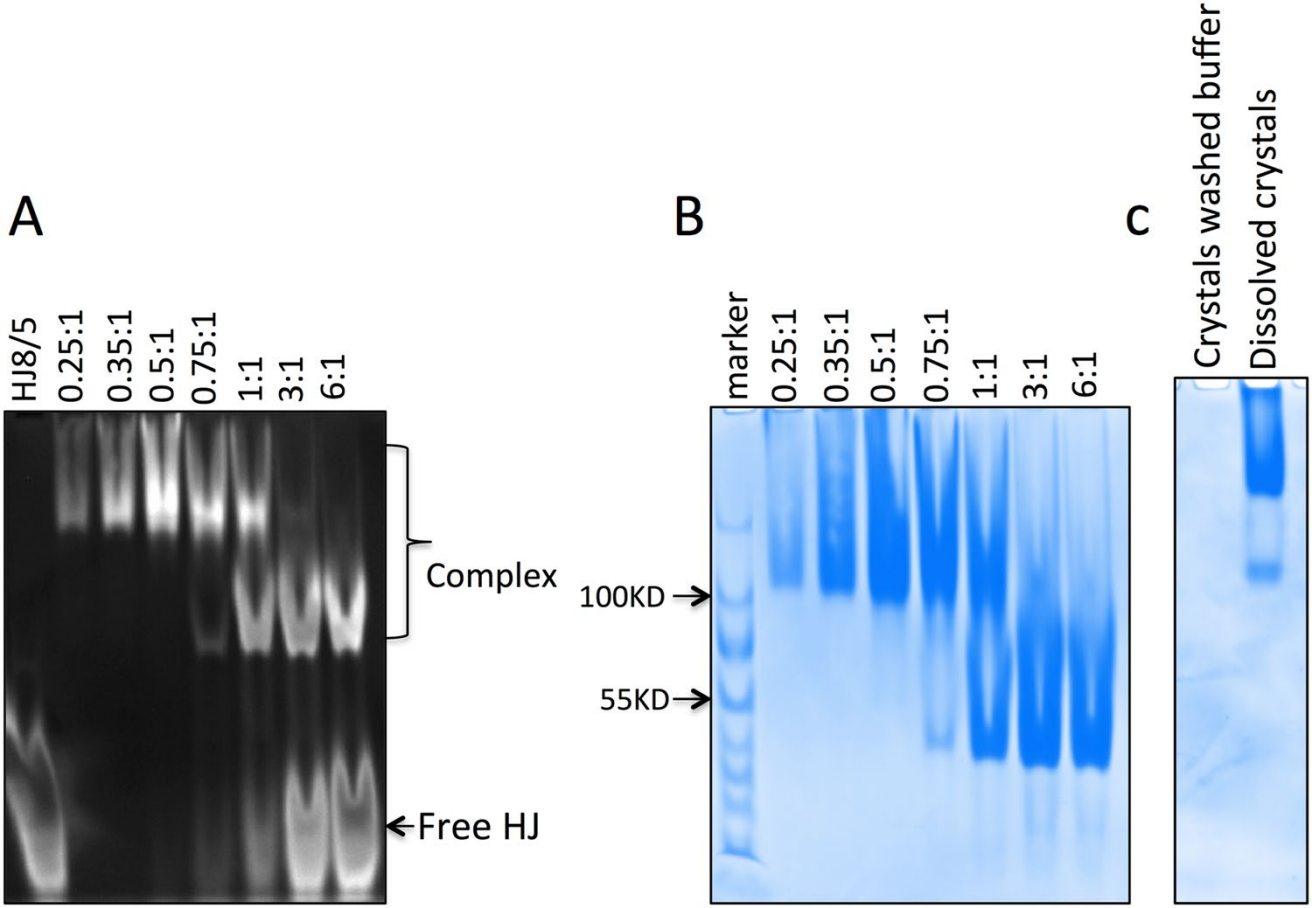
Two distinct complexes of FprC151Stop D135N with HJ8/5. (**A**) Titration of HJ with Fpr. Complexes formed at the HJ: Fpr-dimer ratios of 0.25:1, 0.35:1, 0.5:1, 0.75:1, 1:1, 3:1, 6:1 separated on a native gel, stained with ethidium bromide to visualize DNA. (**B**) The gel above was stained by coomassie blue for protein. (**C**) Dissolved crystals of Fpr-HJ8/5 complex. Crystals extensively washed with well buffer were dissolved and analyzed on a native gel stained by coomassie blue. The final washing buffer had no detectable protein. Original full-length gels for Fig. 4 are shown in Supplementary Figure S9.

To test whether this unique mode of HJ DNA interactions by Fpr could take place in solution, we analyzed the titration of HJ8/5 with Fpr by native PAGE (Fig. 4A,B). Depending on the protein/DNA ratio, two distinct protein-DNA complex species were observed. The lower band shows the apparent molecular mass of ~50 KDa and likely contains one HJ bound to a Fpr dimer. This is consistent with the canonical mode of association of HJ resolvase with DNA, as observed in the structure of RuvC-HJ DNA complex^14^. Upon further addition of Fpr, a more proteinaceous upper band of an apparent mass of ~100 kDa became more intense and this is the only observed complex at the Fpr monomer: HJ ratio of 4:1. We also analyzed dissolved crystals of the Fpr D135N - HJ8/5 complex by native PAGE, and observed the upper band as the main product (Fig. 4C). The larger Fpr-HJ complex formed at higher protein over DNA ratios could be the novel “sandwich”complex observed as the basic building unit in the crystal lattice (Fig. 3B).

### The two forms of Fpr-HJ complexes are in dynamic exchange in solution

**Figure 5:**
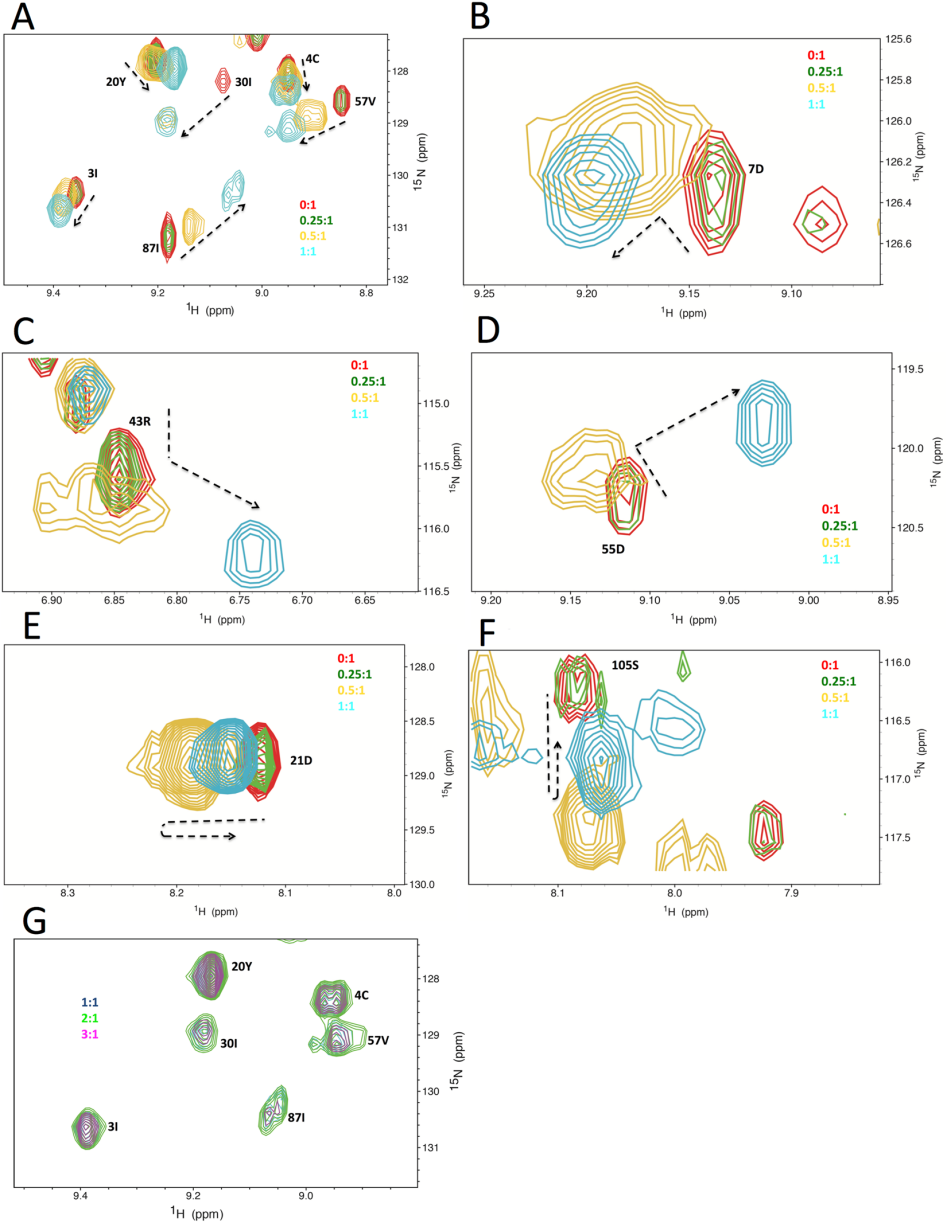
NMR chemical shift perturbation analyses of the interaction between Fpr C151Stop D135N and HJ8/5 in solution. Titration of HJ DNA into Fpr dimer at their molar ratios of 0:1 (red), 0.25:1 (green), 0.5:1 (orange) and 1:1 (cyan). (**A**) A region of the ^1^H-^15^N TROSY-HSQC spectra with residues showing linear chemical changes. (**B**–**F**) Different regions of the spectra with residues that showed nonlinear trajectories of peak shifts. The paths of peak shifts are indicated by dotted arrows. (**G**) Same region as in A, showing further titration of HJ DNA into Fpr dimer at ratios of 1:1 (blue), 2:1 (green), 3:1(magenta).

To further investigate the interactions between HJ and Fpr in solution, we used NMR spectroscopy. Chemical shift assignment for backbone atoms of Fpr C151Stop D135N (Supplementary Figure S5A) was done using the following standard NMR experiments: [^2^H, ^15^N, ^13^C]-TROSY-based HSQC, HNCA, HNCO, HN (CO) CA, HN (CA) CB, and HNCACO. 82% of the total backbone atoms were assigned. Most unassigned residues, due to missing and/or exchange-broadened NMR peaks, were located within surface loops or the αD helix based on the structure of Apo Fpr, suggesting that they are flexible. The key surface-exposed residues involved in DNA interaction were mapped by the chemical-shift perturbations (CSPs) of [^2^H, ^15^N]-TROSY-HSQC spectrum of Fpr upon titration with HJ8/5 DNA (Supplementary Figure S5B-E). When the ratio of HJ to Fpr dimer was above 1:1 (HJ in molar excess), the chemical shift of each residue did not change significantly, indicating that the binding of Fpr dimer and HJ had been already saturated (Fig. 5G). This is consistent with the native gel results where the lower band was the dominant product when the molar ratio of HJ8/5 DNA to Fpr dimer was beyond 1:1, which suggested a stable complex formation between one Fpr dimer with a HJ (2:1 Fpr monomer to HJ). At the ratios below 1:1, significant and gradual chemical shift changes were observed in many residue peaks (Fig. 5A–F and Supplementary Figure S5B). Importantly, while many of the residues showed linear chemical shift changes (Fig. 5A), some residues, including D7, D21, R43, D55 and S105, showed nonlinear trajectory of peak shifts (Fig. 5B to F). This indicates that there were more than one binding modes between protein and DNA in the system. Notably, a number of peaks disappeared due to significant broadening during titration, including those corresponding to residues at the N-terminus of αB near the dimer interface. Peaks corresponding to residues from R89 to I110 also disappeared, except G95, G96, and R99, which show large CSPs (Supplementary Figure S5C–E). These residues are located at the C-terminus of β5, N-terminus of αC, and the intervening loop.

CSPs identified general contact interfaces of protein in HJ-binding, but were insufficient to define the relative orientations of the protein and DNA within the complex. We therefore investigated paramagnetic relaxation enhancement (PRE) effects on [^2^H, ^15^N]-Fpr D135N binding with site-specifically spin-labeled HJ8/5. The method utilizes the fact that the unpaired electron of the spin-label induces line broadening of residues within ~25 Å proximity (Supplementary Tables S4 and S5)^25–28^. We probed with 2 labeling sites by introducing deoxy-4-thiouracil either at the 5′ terminus of HJ8/5-1 (dU1) or in the -TTT- loop region of HJ8/5-1 (dU2) (Supplementary Figures S6 and S7). Deoxy-4-thiouracil was reacted with 3-(2-iodoacetamido)-proxyl to achieve covalent modification with a spin-label (Oxidized state), and the covalent bond was destroyed by adding excess sodium hydrosulfite (Reduced state) for controls. Comparing the [^1^H, ^15^N] TROSY-HSQC spectra for oxidized and reduced conditions, each residue peak was evaluated for the ratio of intensities (heights) of oxidized (*I*_ox_) and reduced (*I*_red_) states. Due to the presence of multiple Fpr molecules that contribute to the same HSQC peak but may be affected differently by the spin label, we did not attempt to use PRE data as distance restraints to derive a solution structure. Instead, the data were interpreted only semi-quantitatively. Residues whose peaks are broadened (0 < *I*_ox_/*I*_red_ < 0.8) but still detectable in the oxidized spectrum, were considered to be in a distance range of 12 Å to 25 Å from the paramagnetic spin-label, whereas residues whose peaks disappeared in the oxidized spectrum were considered to be closer, in at least one of the Fpr molecules. When the spin-label was at the 5′ terminus (dU1), many peaks showed severe broadening and overlapping. Nonetheless, 29 clearly resolved residues were identified and assigned, including 15 residues with peak intensity reduced significantly, suggesting that these residues were near the 5′ end of the HJ8/5-1 strand. For spin-label at dU2, 38 residues were identified and assigned. Of these the peak intensities of 22 residues were significantly decreased, indicating that these residues are proximal to the TTT-loop (Supplementary Tables S4 and S5).

To test whether the PRE effects can be explained by our Fpr-HJ complex crystal structure or a model based on the RuvC-HJ complex, we constructed a “canonical” Fpr-HJ8/5 model based on the RuvC-HJ complex crystal structure^14^. The active Fpr C151stop resolves HJ8/5 by selectively cleaving at 9 bases from the 5′ end (Supplementary Figure S8A), allowing for unambiguous docking of HJ8/5 on the Fpr dimer (Supplementary Figures S6E and S7E). In this model, the two 5 bp arms with the TTT hairpin loop make intimate contacts with Fpr, whereas the N-terminus of the αC helix at the outer edges of the Fpr dimer engages the minor groove of the 8 bp arms. For Du1, all residues that showed significant reduction in peak intensities, with the exception of E145, were more than 30 Å away from the spin label in the RuvC-based model (Supplementary Table S4). Some (S35, N68, Y73, Y80 and N138) of these residues are within 25 Å in at least one of the molecules in our Fpr-HJ complex crystal structure. Other residues do not come within 25 Å of the spin label in either model. Conversely, most peaks that did not undergo significant relaxation (*I*_ox_/*I*_red_ > 0.8) fitted both the Fpr-HJ complex crystal structure and the RuvC-based docking model with distances large than ~25 Å (Supplementary Figure S6). For dU2, many residues that underwent significant reduction in peak intensities were within 25 Å in at least one of the molecules in the Fpr-HJ complex crystal structure, but T23, A31, I32, S114, and Y119 had distances large than 25 Å to the spin label in all molecules (Supplementary Table S4). The RuvC-based model does not account for the PRE effect for most residues. Most of the residues unaffected by dU2 are beyond ~25 Å in either model, although several are within 25 Å in at least one of the molecules in the crystal structure (Supplementary Figure S7). Collectively, neither the RuvC-based “canonical” binding mode, which likely represents the productive conformation for HJ cleavage, or the Fpr-HJ crystal structure alone successfully explained the PRE data. Moreover, even both models combined did not fully account for the PRE data, suggesting that the Fpr-HJ complexes formed in solution could be highly dynamic and heterogeneous.

### DNA branch selectivity of Chimeric RuvC-fowl poxvirus resolvase

Previous studies of Fpr showed that it can form complexes with various kinds of DNA structures, including Holliday junction, Y junction, bulges, but had the highest binding affinity for Holliday junction^9^. The cleavage activity of Fpr was also tested on a variety of branched DNA substrates, showing that the HJ cleavage takes place at 1-nt from the branch point toward the 3′-end of the scissile strand^9^. The HJ cleavage activity of Fpr is not target DNA sequence-dependent, in contrast to that by RuvC where the cleavage preferentially occurs at the (A/T) TT↓(G/C) cognate sequence^15,29^.

In the RuvC-HJ DNA structure, the αB helix at the dimer interface and the preceding loop fit into the central gap of the 4-way junction and make critical DNA contacts^14^. The αB and the preceding loop in Fpr are shorter and thus make a much less pronounced protrusion on the protein surface. It was proposed that the similarly short αB in Cpr could only contact the sugar-phosphate backbone at the junction center of HJ^11^. To gain insights into the HJ cleavage of Fpr without sequence selectivity, a chimeric protein RuvC-fowl poxvirus resolvase (CRFpr) was constructed by replacing Fpr K61~I72 that includes the N-terminal portion of αB and the preceding loop, with the corresponding residues in *Tth*RuvC, E71~A87, based on the structure comparison of Fpr and RuvC. Previous studies showed that mutations of RuvC residues F73, F74, Y75, R76 and K83 affect the binding and cleavage activity greatly^14,16,30^. Thus, the shorter loop of Fpr spanning K61 to I72 may be responsible for its non-specific cleavage activity.

**Figure 6:**
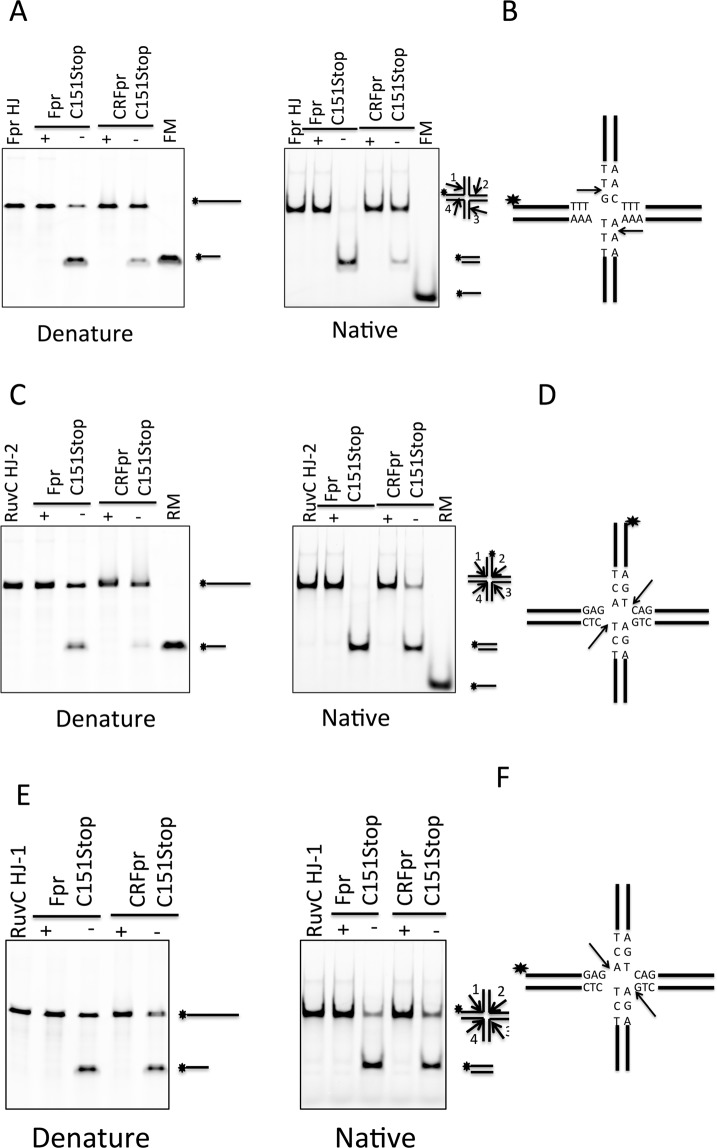
Comparison of the HJ cleavage activities of Fpr C151stop and CRFpr C151 stop. (**A**) Cleavage of FprHJ substrate by Fpr and CRFpr. The reaction mixture (20 μl) containing Fpr dimer (300 nM) or CRFpr dimer (300 nM) with FprHJ (100 nM), was incubated at 37 °C for 10 min. The reaction products were separately analyzed on denaturing (left) and native gels (right). FprHJ, FprHJ alone in reaction buffer as a control. FM, FprHJ marker. (**B**) Central sequences of FprHJ. The star indicates the fluorescently labeled position and arrows indicate the cleavage positions. (**C**) Cleavage of the bimobile RuvCHJ-2 substrate by Fpr and CRFpr. The reaction condition was the same as above. RuvCHJ-2 has 6-FAM label at the 5′ of strand 2. RM, RuvCHJ marker. (**D**) Central sequences of RuvCHJ-2. The star shows the labeled position and arrows indicate the cleavage positions. (**E**) Cleavage of RuvCHJ-1 substrate by Fpr and CRFpr. The reaction condition was the same as above. RuvCHJ-1 has 6-FAM label at the 5′ of strand 1. RM, RuvCHJ marker. (**F**) Central sequences of RuvCHJ-1. The star shows the labeled position and arrows indicate the cleavage positions. Each DNA cleavage experiment was repeated 3 times and a representative gel is shown in the figure. Original full-length gels for Fig. 6 are shown in Supplementary Figure S12.

Exchanging the loop regions did not disrupt the DNA-binding ability of Fpr as demonstrated by the slightly lower Kd observed for CRFpr (Supplementary Table S3). We incubated Fpr and CRFpr with bulge, Y junction and HJ separately (Supplementary Table S2) and the reaction products were analyzed on both native and denaturing PAGE. Fpr cuts bulge and Y junction DNA well, but CRFpr showed much reduced cleavage activity on these substrates (Supplementary Figure S8B, C). CRFpr also showed weaker activity (Fig. 6A,B) on a Holliday Junction substrate ‘Fpr HJ’, which Fpr was previously shown to efficiently resolve by cleavage on the strands 1 and 3 near the immobile junction point^9^. To find out whether this is due to a generally weaker DNA cleavage activity of the chimeric protein CRFpr or because the grafted loop of RuvC has conferred sequence selectivity to Fpr, we also performed cleavage assay of CRFpr with ‘RuvC HJ’ with the fluorescence label either at the 5′ end of strand-1 (RuvCHJ-1) or 5′ end of strand-2 (RuvCHJ-2). RuvC HJ is a ‘bimobile’ junction with TC nucleotides in junction center, which is the preferred target sequence for RuvC that cleaves between T and C on the strand-2^16^. Fpr cuts this ‘RuvC HJ’ well, showing no significant strand bias (compare Fig. 6C and E). CRFpr also resolved the ‘RuvC HJ’ to near completion under the condition tested, generating a strong product band in the native gel. Interestingly however, CRFpr generated only a very faint band in the denaturing gel on the substrate RuvCHJ-2, and a much stronger product band on RuvCHJ-1. Thus, CRFpr has a clear preference to resolve the RuvC HJ by cleaving the strands 1 and 3, opposite that of RuvC. These results suggest that the unique loop K61~I72 of Fpr is important for broad substrate specificity and sequence-independent HJ resolution of Fpr. Corresponding RuvC loop E71~A87 makes direct base contacts near the HJ junction and thus confers higher DNA structure and sequence selectivity, but it does so in a context-dependent fashion and when grafted onto Fpr, it converted Fpr into a HJ resolvase with a novel sequence selectivity.

## Discussion

The DNA resolvases orthologous to the A22 protein of vaccinia virus play an essential role in poxviral replication, by resolving HJ structures formed at the inverted repeat junctions in a concatemer of linear genomic DNAs. Our crystal structure of Fpr and the recently published structure of Cpr^11^ confirm that these enzymes have the overall fold similar to that of the bacterial RuvC resolvase and the yeast mitochondrial Ydc2. However, the poxvirus resolvases have unique features as well, including the flatter DNA-binding surface across the dimer interface attributed to the shorter αB helix, the protrusion made by the longer helix αC at the distal edges of the Fpr dimer, and the constriction of the Fpr dimer due to the short helix αA that generates basic side grooves near the dimer interface. Our studies show how these structural features contribute to the non-canonical mode of Fpr-HJ interaction and could account for the promiscuous DNA-binding and branch cleavage activities of Fpr.

The crystal structure of the Fpr-HJ complex showed a novel mode of resolvase-HJ interaction distinct from that observed in the RuvC-HJ complex. In this binding mode, the -TTT- hairpin loops of HJ8/5 fit into the side groove of Fpr, where the unpaired thymine bases make interactions with αB and the preceding loop. In the canonical RuvC-HJ complex, the longer αB at the dimer interface docks into the junction center of the unstacked HJ DNA. In the Fpr-HJ complex, the long αC helix at the outer edge of the dimer and the preceding loop instead make backbone contacts with the crossover junction to maintain the stacked conformation of HJ. The native gel analysis and NMR data support that this and potentially other non-canonical complexes form in solution and are in equilibrium with the canonical complex, highlighting the versatility of Fpr in binding various DNA structures. It is possible that these interactions help Fpr engage branched DNA structures other than the canonical HJ substrate. The unique mode of association with the short hairpin loop might also help association of Fpr with particular regions of poxviral genome, possibly in the vicinity of hairpin telomeres or its replication intermediates.

By generating a chimeric resolvase we showed that the region including the N-terminus of the short αB helix and the corresponding ‘flat’ DNA-binding surface across the dimer interface is important in the promiscuous and sequence-independent DNA cleavage by Fpr. The F64A substitution had less severe effect on HJ resolution activity of Fpr compared to some other mutations within the DNA-binding cleft such as K129A (Fig. 2). Thus, the short loop spanning K61 to I72, which is involved in unique DNA-binding modes of Fpr (Fig. 3 and Supplementary Figure S4), may not make specific DNA contacts required for HJ resolution. Interestingly, substituting the N-terminal portion of the longer αB loop and the preceding loop from RuvC made Fpr more sequence-selective, while conferring target sequence preference distinct from that of RuvC. It is possible that the chimeric enzyme CRFpr mimics the general mode of HJ-DNA binding by RuvC, but the grafted loop makes unique interactions with nucleobases flanking the junction center. The residues E71~A87 of T.th. RuvC rich in aromatic residues is stabilized by an extensive hydrogen-bond network with surrounding residues^16^. Thus, the precise conformation of these residues and the DNA-interactions are likely context-dependent, which could have led to the generation of a DNA resolvase with novel sequence selectivity.

In conclusion, our unique Fpr-DNA complex crystal structure and corroborating solution NMR and biochemical experiments provide insights into the mechanism of broad DNA substrate specificity of Fpr, which may facilitate further functional studies of fowlpox virus and the development of anti-poxviral strategies.

## Methods

### Protein preparation

A codon-optimized synthetic gene of Fpr was inserted into pET24a vector between the NdeI and BamHI enzyme sites. To express C151stop, we subsequently mutated the C151 codon to a stop codon. All other mutants were generated in the background of C151stop, using the Quick-change site-directed mutagenesis procedure. *E. coli* strain BL21 (DE3) transformed by each plasmid was grown in 4 L of LB medium. When OD_600_ reached ~0.5, isopropyl-β-D-thio-galactoside (IPTG) was added to a final concentration of 1 mM to induce protein expression. The cells were further cultured at 20 °C for 18 h and then harvested by centrifugation. The collected cells were re-suspended in 100 ml buffer A (20 mM Tris-HCl, pH 7.4, 0.25 M NaCl and 5 mM 2-mercaptoethernal), disrupted by sonication, and spun down at 59,000 g for 1 h. The cleared lysate was filtered through a surfactant-free cellulose acetate membrane with a 0.2 μm pore-size and loaded on the Hi-trap Heparin-Sepharose column, which was pre-equilibrated with buffer A. Protein was eluted by a linear NaCl concentration gradient from 0.25 to 1.5 M. The peak fractions were concentrated and used for crystallization and biochemical studies.

### Crystallization

For the apo Fpr crystal, oligonucleotides HJ13/13-1, HJ13/13-2, HJ13/13-3 and HJ13/13-4 (Supplementary Table S1) were mixed at equimolar ratio in annealing buffer C (10 mM Tris-HCl pH 7.4, 150 mM NaCl), heated to 373 K for 3 min, and slowly cooled to room temperature for making DNA Holliday Junction HJ13/13. The Fpr mutant C151Stop D135N K102A K103A Q104A K65A (7.3 mg/ml) was mixed with HJ13/13 at a 1:1.2 molar ratio (Fpr-dimer: HJ) then dialyzed against buffer D (10 mM Tris-HCl pH 7.4, 150 mM NaCl, 1 mM Tris (2-carboxy ethyl) phosphine hydrochloride) overnight at room temperature. The complex thus formed was mixed with an equal volume of the reservoir solution 0.1 M Bis-Tris-HCl buffer (pH 6.5), 10 mM CdCl_2_, 6% (w/v) PEG3350 and equilibrated via the hanging drop vapor diffusion method at 20 °C. Crystals were obtained by seeding with smaller crystals initially obtained in the same condition, and soaked in a reservoir solution containing 25% (w/v) ethylene glycol for cryoprotection. The content of the crystals was analyzed on a 15% TBE-urea gel and 6% DNA retardation gel (Invitrogen). We did not obtain any of apo protein crystals without DNA. For phasing, the crystals were soaked with 0.1 mM HgCl_2_ overnight before being flash frozen in liquid nitrogen.

For Fpr complex with HJ DNA with two TTT hairpins, oligonucleotides HJ8/5-1 and HJ8/5-2 were annealed as above for preparing HJ8/5 (Supplementary Table S1). The Fpr mutant C151Stop D135N (7.3 mg/ml) was mixed with HJ8/5 at a 1:1.2 molar ratio then dialyzed against the buffer D overnight at room temperature. The protein-DNA complex was crystallized similarly to the apo protein above, using a reservoir solution containing 0.1 M sodium cacodylate pH 6.6, 150 mM NaCl at 20 °C and soaked in the reservoir solution supplemented with 25% (w/v) ethylene glycol for cryoprotection. The content of the crystals was also analyzed on a 15% TBE-Urea gel and a 6% DNA retardation gel (Invitrogen) separately. For phasing, the crystals were soaked in 0.35 mM CH_3_HgCl overnight.

### Data collection and structural determination

All x-ray diffraction data from the native or heavy atom derivative crystals, were collected at the Advanced Photon Source NE-CAT Beamlines 24-ID-C and 24-ID-E and at the wavelength of 0.979 Å. Datasets were processed using HKL2000^31^ or XDS^32^. Both structures were solved by the single-wavelength anomalous dispersion (SAD) method using Hg anomalous signals. SHELX C/D/E^33^ software located the Hg atoms and generated electron density maps with clearly recognizable protein or DNA structural features. Molecular replacement was done using Molrep^34^ to place the initial model, obtained by pruning loops off the RuvC structure with most of the secondary structure elements kept, into the SAD electron densities. Subsequent iterative model building using COOT^35^ and phase-restrained refinement with NCS restraints and simulated-annealing using PHENIX suite^36^ further improved the maps. Further refinement and model building using higher-resolution native data resulted in the final R_work_/R_free_ values of 27.1/27.9% and 21.0%/26.8% for the APO and complex structure, respectively. The complex contains one copy of DNA Holliday junction and four copies of Fpr monomers in an asymmetric unit. The summary of data collection and refinement statistics is shown in Table 1. The molecular graphics images were produced using PyMOL (www.pymol.org) and CCP4MG^37^. The coordinates and reflection data for APO Fpr and Fpr-HJ complex have been deposited in the RCSB Protein Data Bank with accession code 6P7A and 6P7B, respectively.

**Table 1:** Data collection and refinement statistics.

	APO Fpr	Fpr-HJ complex
**Data collection**		
Wavelength (Å)	0.979	0.979
Resolution range (Å)	19.8 - 3.1 (3.2 - 3.1)	39.8 - 3.3 (3.4 - 3.3)
Space group	P3_2_	P4
**Unit cell**		
a, b, c (Å)	82.62 82.62 68.76	143.51 143.51 56.54
α, β, γ (º)	90 90 120	90 90 90
Total reflections	24498 (2480)	62647 (6167)
Unique reflections	9345 (940)	17192 (1669)
Multiplicity	2.6 (2.6)	3.6 (3.7)
Completeness (%)	96.30 (97.72)	98.27 (97.02)
*I/σ(I)*	13.1 (2.2)	8.2 (1.2)
R-merge (%)	3.00 (42.4)	5.39 (94.7)
R-meas (%)	3.73 (53.4)	6.29 (111)
R-pim (%)	2.19 (32.0)	3.19 (56.9)
CC1/2	1.0 (0.80)	1.0 (0.68)
**Refinement**		
Reflections/#R-free	9343/437	17192/1658
R-work/R-free (%)	27.1/27.9	21.0/26.8
# of non-hydrogen atoms	2337	6001
Protein	2384	4857
DNA	N/A	1144
Ligands	6	—
Protein residues	289	591
**r.m.s.deviation**		
Bond lengths (Å)	0.002	0.003
Bond angles (º)	0.52	0.66
**Ramachandran plot**		
Favored (%)	95.70	90.39
Allowed (%)	4.30	9.61
Outliers (%)	0.00	0.00
Average B-factor	155.06	219.00
Protein	154.87	215.49
DNA	N/A	233.89
Ligands	230.61	

Statistics for the highest-resolution shell are shown in parentheses.

### NMR spectroscopy

The ^2^H, ^15^N, ^13^C-labeled Fpr C151Stop D135N protein was obtained by growing the transformed BL21(DE3) bacterial cells in M9/D_2_O minimal medium with a final pH of 8.2 (50 mM Na_2_HPO_4_, 25 mM KH_2_PO_4_, 10 mM NaCl, 5 mM MgSO_4_, 0.1 mM CaCl_2_, 1% Thiamine, 0.15% ^15^NH_4_Cl, 0.3% Deuterated ^13^C_6_ D-glucose). An overnight LB culture was harvested and resuspended in a M9/H_2_O medium, and grown at 37 °C until OD_600_ reaches ~0.5. Cells were collected again and resuspended in 50 mL M9/D_2_O, containing 1.5 g/L ^15^NH_4_Cl (Cambridge isotope lab.) and 2 g/L ^13^C_6_ D-glucose (Cambridge isotope lab.) as the sole nitrogen and carbon source, respectively. At OD_600_ of ~0.5, the cells were transferred to 1 L culture of M9/D_2_O, grown to OD_600_ of ~0.9 and cooled down to 20 °C. IPTG was added to a final concentration of 1 mM for induction of protein expression. The cells were further cultured at 20 °C for 24 h and then harvested by centrifugation. The proteins were purified as described above.

All NMR data were collected on Bruker 900 MHz spectrometer equipped with a 5 mm TCI cryoprobe at 303 K (NMR center at the University of Minnesota) on ~600 μM protein samples in buffer E (25 mM L-Arg and 2 5mM L-Glu)^38^. All data were processed using NMRpipe^39^, and visualized using CcpNMR^40^. 2D ^1^H-^15^N correlation spectra were obtained using the transverse relaxation-optimized spectroscopy and heteronuclear single-quantum coherence (TROSY-HSQC) experiments^41,42^ with chemical shifts referenced to 4,4-dimethyl-4-silapentane-1-sulfonic acid (DSS). Backbone assignments were achieved using [^2^H, ^15^N, ^13^C]-TROSY-based HSQC, HNCA, HNCO, HN (CO) CA, HN (CA) CB, and HNCACO. The surface residues that interacted with DNA HJ were mapped based on the analyses of chemical shift perturbations (CSPs). In short, HJ8/5 (Supplementary Table S1) was titrated into protein gradually with the DNA: Fpr (dimer) ratios of 0:1, 0.25:1, 0.35:1, 0.5:1, 0.75:1, 1:1, 2:1 and 3:1. At each ratio TROSY-HSQC spectrum was measured. Residues whose chemical shift changed gradually or peak disappeared were identified. The magnitudes of CSPs were calculated using amide ^1^H and ^15^N chemical shifts according to the following equation: ∆δ = (∆δ_H_^2^ + 0.154∆δ_N_^2^)^1/2^, where ∆δ_H_ and ∆δ_N_ denote ^1^H and ^15^N chemical shift change, respectively, and 0.154 is a scaling factor (33). All 3D spectra were typically collected with 8-32 scans, depending on sample concentration and experiment sensitivity, with 2048 (proton), 40–64 (nitrogen), 80–128 (carbon) points. All TROSY-HSQC with ^2^H and ^15^N labeling experiments were acquired with 24 scans, 2048 (proton) and 128 (nitrogen) data points.

The interaction of Fpr mutant C151Stop D135N with HJ8/5 was also probed using paramagnetic spin labeling (22, 24). In short, HJ8/5 with two positions modified with deoxy-4-thiouracil separately were purchased (Midland Certified Reagent Company) and reacted with 3-(2-iodoacetamido)-proxyl (Sigma) overnight to achieve covalent modification for making dU1 HJ8/5 and dU2 HJ8/5. Protein dimer and the spin-labeled HJ8/5 (Supplementary Table S1) were mixed at 1:1.5 molar ratio and then used for TROSY-HSQC experiments. After that, 5 mM sodium hydrosulfate was added to the same tube to break the covalent bond of deoxy-4-thiouracil and 3-(2-iodoacetamido)-proxyl and then samples were used for TROSY-HSQC experiments immediately. Residues in proximity of the spin labels were identified through comparing the peak heights in the two spectra before (Oxidized state) and after (Reduced state as control) sodium hydrosulfate added (*I*_ox_/*I*_red_). Specifically for dU1, the sodium hydrosulfite treatment caused some protein to precipitate, leading to overall (approximately by a factor of 1.5~2.0) loss of intensity in the reference spectrum. To correct for this, *I*_ox_/*I*_red_ for each residue was normalized against the average *I*_ox_/*I*_red_ of several isolated residues (I2, I3, F18, Y20 and L147) unlikely to be involved in DNA-binding based on the CSP experiment.

### RuvC-like Fpr-HJ docking model

The RuvC HJ-based canonical Fpr-HJ complex was manually modeled by superimposing an Fpr dimer onto the RuvC-HJ complex (PDB ID: 4LD0)^14^ and geometry minimized by molecular dynamics in SCHRODINGER Suite (Prime X, Schrödinger, LLC, New York, NY, 2017). The orientation of the HJ8/5 DNA was determined based on the cleavage pattern (Supplementary Figure S8A) to position the scissile phosphate 9 bases from the 5′ terminus in the active site.

### DNA binding activity by fluorescence polarization

The oligonucleotides (IDT) FHJ13/13-1 labeled at the 5′ end with 6-carboxyfluorescein (6-FAM) and FHJ13/13-2 were mixed in annealing buffer C (Supplementary Table S1) and were heated to 373 K for 3 min and slowly cooled to room temperature for making F13/13HJ. Purified proteins were dialyzed into buffer F (25 mM Tris-HCl pH 7.4, 100 mM NaCl, 5 mM EDTA pH8.0, 1% Glycerol) at 4 °C overnight, and the concentrations were measured by UV absorption. Protein concentration was expressed as that of a dimer. The indicated amount of mutants were mixed with F13/13HJ (20 μl total) at the final DNA concentration of 10 nM, then 15 μl was loaded on each well of a 384 well plate (Corning 3821BC), and incubate at 37 °C for 20 min (13). Positive reference samples contained all components except the enzyme. Binding was monitored through measuring change of Fluorescence Polarization (FP) using BioTek Synergy^TM^2 Microplate reader. Data were analyzed by Origin software and fit to the formula: y = 1/(1 + (K/(x − 0.5*(A + x + K − ((A + x + K)^2–4^ * A * x)^0.5^)))) (23).

A: DNA concentration (10 nM); x: protein concentration. y: bound fraction; K: dissociation constant (Kd).

### HJ DNA cleavage assay

The 5′ 6-FAM-labeled strands (IDT) and unlabeled strands were annealed in buffer C (Supplementary Table S2) by heating to 373 K for 3 min and slow-cooling to room temperature. Cleavage assays (20 μl mixture) were carried out in buffer G (25 mM Tris-HCl pH 7.4, 100 mM NaCl, 5 mM 2-mercaptoethanol, 5% glycerol, 100 μg mL^-1^ BSA). 5 mM EDTA was added for negative controls (lanes labeled “+” in figures) whereas 5 mM MgCl_2_ was added for testing cleavage activity (lanes labeled “−”). The indicated amount of mutant proteins (300 nM or 600 nM final concentration, protein concentration was calculated as dimer) mixed with HJ (100 nM final concentration), incubated at 37 °C for 10 mins or 1 hour and stopped with stop solution (2.5% SDS, 100 mM EDTA, final concentrations). Products of the reaction were analyzed by 15% TBE-Urea gel and 6% DNA retardation gel separately (14,15). The images were scanned using Typhoon FLA 9500 and the data analyzed using ImageJ. Each DNA cleavage experiment was performed 3 times and a representative gel is shown in the figures. Full-length gel images are shown in Supplementary Figures S9, S10, S11, and S12, to support figures with cropped gel images.

## Supplementary information

Supplementary Information.

## Data Availability

The coordinates and reflection data for APO Fpr and Fpr-HJ complex have been deposited in the RCSB Protein Data Bank with accession code 6P7A and 6P7B, respectively. NMR chemical shift assignments for Fpr have been deposited in the Biological Magnetic Resonance Data Bank (BMRB) under accession number 50141.
