# Supplementary material for: Structural insights into the promiscuous DNA binding and broad substrate selectivity of fowlpox virus resolvase
Source: Sci Rep. 2020 Jan 15;10:393. doi: 10.1038/s41598-019-56825-w (PMC6962361; doi:10.1038/s41598-019-56825-w)
Supplement: Supplementary file 1 — Supplementary Information. [file 41598_2019_56825_MOESM1_ESM.pdf]

Supplementary information

Structural insights into the promiscuous DNA binding and broad substrate selectivity of fowlpox virus resolvase

Na Li[1#], Ke Shi[1], Timsi Rao[1], Surajit Banerjee[2], Hideki Aihara[1*]

[1] Department of Biochemistry, Molecular Biology, and Biophysics, University of Minnesota, 6-155 Jackson Hall, 321 Church Street S.E., Minneapolis, MN 55455, USA

[2] Northeastern Collaborative Access Team, Cornell University, Advanced Photon Source, Lemont, Illinois, USA, 60439

[#] Current address: College of Biological Engineering, Henan University of Technology, Zhengzhou, 450001, People's Republic of China

[*] Corresponding author, email: aihar001@umn.edu, Phone: +1-612-624-1491

Supplementary Table S1. Oligonucleotides used for crystallization, NMR, and binding assay

HJ13/13
HJ13/13-1: 5'-CGAGAATTCCGGATTAGGGATCCGCG-3'
HJ13/13-2: 5'-CGCGGATCCCTAAGCTCCATCGATCG-3'
HJ13/13-3: 5'-CGATCGATGGAGCCGCTAGGCCTCCG-3'
HJ13/13-4: 5'-CGGAGGCCTAGCGTCCGGAATTCTCG-3'

HJ8/5(1)
HJ8/5-1: 5'-ATCGTCGGGGAAGTTTCTTCCTGAGTTGA-3'
HJ8/5-2: 5'-TCAACTCAACTCGTTTCGAGTCCGACGAT-3'

FHJ13/13
FHJ13/13-1:
FAM6-5'-
CGGAGGCCTAGCGTCCGGAATTCTCGTTTCGAGAATTCCGGATTAGGGATCCGCG-3'
FHJ13/13-2:
5'-CGCGGATCCCTAAGCTCCATCGATCGTTTCGATCGATGGAGCCGCTAGGCCTCCG-3'

dU1-HJ8/5
dU1-HJ8/5-1: 5'-(4-thio-dU)ATCGTCGGGGAAGTTTCTTCCTGAGTTGA-3'
dU1-HJ8/5-2: 5'-TCAACTCAACTCGTTTCGAGTCCGACGAT-3'

dU2-HJ8/5
dU2-HJ8/5-1: 5'-ATCGTCGGGGAAG(4-thio-dU)TTCTTCCTGAGTTGA-3'
dU2-HJ8/5-2: 5'-TCAACTCAACTCGTTTCGAGTCCGACGAT-3'

Supplementary Table S2. Oligonucleotides used for cleavage assay

FHJ8/5
FHJ8/5-1: 6FAM-5'-ATCGTCGGGGAAGTTTCTTCCTGAGTTGA-3'
HJ8/5-2: 5'-TCAACTCAACTCGTTTCGAGTCCGACGAT-3'
FHJ8/5 Marker: 6FAM-5'-ATCGTCGGG 9nt

Holliday junction For Fpr (FprHJ)(2)
FprHJ strand 1:
6FAM-5'-CCACCAGAAACACGCCACAGTTTTTGTTTTGATTGCGAGGCCGTCCTACC-3'
FprHJ strand 2:
5'-GGTAGGACGGCCTCGCAATCAAAACTTTTTGAGCACGCGAGATGTCAACG-3'
FprHJ strand 3:
5'-CGTTGACATCTCGCGTGCTCAAAAAAAAAACAGATGCGGAGTGAAGTTCC-3'
FprHJ strand 4:
5'-GGAACTTCACTCCGCATCTGTTTTTAAAAACTGTGGCGTGTTTCTGGTGG-3'
FprHJ Maker: 6FAM-5'-CCACCAGAAACACGCCACAGTTTTTGT-3' 27nt

Bulge(2)
Bulge strand 1:
6FAM-5'-CCACCAGAAACACGCCACAGTTTTTGTTTTGATTGCGAGGCCGTCCTACC-3'
Bulge strand 2:
5'-GGTAGGACGGCCTCGCAATCTTTTGTTTTTCTGTGGCGTGTTTCTGGTGG-3'
Bulge Marker: 6FAM-5'-CCACCAGAAACACGCCACAG 20nt

Y junction(2)
Y junction strand 1:
5'-CCACCAGAAACACGCCACAGTTTTTGTTTTGATTGCGAGGCCGTCCTACC-3'
Y junction strand 2:
6FAM-5'-GGTAGGACGGCCTCGCAATCAAAACTTTTTGAGCACGCGAGATGTCAACG-3'
Y junction strand 3:
5'-CGTTGACATCTCGCGTGCTCAAAAAAAAAACTGTGGCGTGTTTCTGGTGG-3'
Y junction Marker: 6FAM-5'-GGTAGGACGGCCTCGCAATCAAAAC-3' 25nt

Holliday junction For RuvC (RuvCHJ)(3)
RuvCHJ stand 1:
5'-ATCCTCTAGACAGCTCCATGGCATCTGCCGAGACTGGCTGTGGCTAGCAAGGCACT
GGTAGAAT-3'
RuvCHJ strand 2:
5'-ATTCTACCAGTGCCTTGCTAGCCACAGCCAGTCAGCCGATTGCGGGACATCTTTGC
CCACCTGC-3'
RuvCHJ strand 3:
5'-GCAGGTGGGCAAAGATGTCCCGCAATCGGCTGAGACCGAGCACGATCTGTTGTAATC
GTCAAGC-3'
RuvCHJ strand 4:

5'-GCTTGACGATTACAACAGATCGTGCTCGGTCTCTCGGCAGATGCCATGGAGCTGTCT
AGAGGAT-3'

RuvCHJ-1
RuvCHJ-1 stand 1:
6FAM-5'-
ATCCTCTAGACAGCTCCATGGCATCTGCCGAGACTGGCTGTGGCTAGCAAGGCACTGGT
AGAAT-3'
RuvCHJ strand 2:
5'-ATTCTACCAGTGCCTTGCTAGCCACAGCCAGTCAGCCGATTGCGGGACATCTTTGCC
CACCTGC-3'
RuvCHJ strand 3:
5'-GCAGGTGGGCAAAGATGTCCCGCAATCGGCTGAGACCGAGCACGATCTGTTGTAAT
CGTCAAGC-3'
RuvCHJ strand 4:
5'-GCTTGACGATTACAACAGATCGTGCTCGGTCTCTCGGCAGATGCCATGGAGCTGTC
TAGAGGAT-3'

RuvCHJ-2
RuvCHJ stand 1:
5'-ATCCTCTAGACAGCTCCATGGCATCTGCCGAGACTGGCTGTGGCTAGCAAGGCAC
TGGTAGAAT-3'
RuvCHJ-2 strand 2:
6FAM-5'-
ATTCTACCAGTGCCTTGCTAGCCACAGCCAGTCAGCCGATTGCGGGACATCTTTGCCCAC
CTGC-3'
RuvCHJ strand 3:
5'-GCAGGTGGGCAAAGATGTCCCGCAATCGGCTGAGACCGAGCACGATCTGTTGTAA
TCGTCAAGC-3'
RuvCHJ strand 4:
5'-GCTTGACGATTACAACAGATCGTGCTCGGTCTCTCGGCAGATGCCATGGAGCTGT
CTAGAGGAT-3'
RuvCHJ Marker: FAM6-5'-ATTCTACCAG TGCCTTGCTA GCCACAGCCAGT-3'   32nt

Supplementary Table S3. HJ DNA binding affinity of mutant Fpr proteins

Protein	Kd (±Std. Error) (nM)
Fpr	167.61±61.59
K11A	252.06±83.15
N12A	159.87±17.59
I30A	111.90±39.83
E33A	130.90±45.49
K34A	301.25±107.85
S35A	141.80±47.64
Q62A	101.75±25.86
F64A	108.97±33.59
K65A	128.39±65.83
S66A	183.50±71.69
N68A	32.90±30.71
K70A	224.21±12.97
T92A	114.87±33.95
F93A	139.75±148.49
K94A	85.84±31.59
G95A	87.02±34.75
S97A	78.69±47.75
R99A	98.21±50.10
R101A	16.64±8.48
K103A	122.70±44.00
S105A	145.05±37.05
D107A	98.85±37.97
K126A	61.05±20.12
K129A	83.95±35.31
D135N	132.94±35.62
CRFpr	53.29±120.36

Fpr: Fpr C151Stop as the wild type; The mutations were introduced in the background of Fpr C151Stop. CRFpr: CRFpr C151Stop

Supplementary Table S4. Residues whose peaks became broadened or disappeared in paramagnetic relaxation enhancement (PRE) experiment with the dU1 or dU2 label

	dU1 HJ8/5			dU2 HJ8/5	
Residues	I_{ox}/I_{red}	Distance 1/2 (Å)	Residues	I_{ox}/I_{red}	Distance 1/2 (Å)
T23	0.81	41.7/42.0	T23	0.76	26.1/41.4
S35	0.52	23.0/41.4	A31	0.79	32.2/30.2
R51	0.50	30.8/47.3	I32	0.36	27.9/26.9
N68	0.53	16.8/34.3	T38	0.22	7.8/12.8
Y73	0.56	13.4/35.4	A46	0.79	18.9/25.4
Y80	0.80	26.7/46.1	D55	0.75	18.3/31.8
N83	0.76	33.5/45.2	Y73	0.71	14.7/18.3
S114	0.46	38.3/31.7	F78	0.78	19.3/27.7
N138	0.80	25.8/28.2	Y80	0.68	14.5/23.1
G140	0.82	27.1/34.1	S82	0.75	21.3/30.3
L141	0.77	29.1/31.1	N83	0.75	18.8/32.0
E145	0.79	30.4/24.2	T84	0.66	16.6/29.3
L148	0.77	33.3/30.4	Y119	0.80	37.5/35.4
I87	N/A[a]	22.8/32.8	N138	0.70	23.8/29.1
T38	N/A[a]	17.0/43.3	G140	0.64	22.6/33.4
			L141	0.68	23.8/33.9
			S146	0.73	15.3/28.0
			L147	0.78	16.5/29.7
			L148	0.70	20.4/30.9
			I87	N/A[a]	13.4/24.7
			S35	N/A[a]	17.5/17.6
			S114	N/A[a]	36.8/37.6

[a] The peaks for these of residues in the oxidized spectrum are too broad to be observed.

I_{ox}/I_{red}: Relative peak intensity (height) between the oxidized (I_{ox}) and reduced (I_{red}) spectra.

Distance: The closest distance between the labeled site of dU1 or dU2 and the indicated residue in the Fpr-HJ complex crystal structure as shown in Figure 3F (labeled as 1) and in the RuvC-based docking model of Fpr-HJ complex as shown in Supplementary Figures S6E and S7E (labeled as 2). The distances that do not fit the PRE data are highlighted in red.

Supplementary Table S5. Residues whose peak intensity did not change significantly in PRE experiment with the dU1 or dU2 label

dU1 HJ8/5			dU2 HJ8/5		
Residues	I_{ox}/I_{red}	Distance 1/2 (Å)	Residues	I_{ox}/I_{red}	Distance 1/2 (Å)
I2	1.00	32.9/43.8	I2	0.93	25.5/36.4
I3	0.92	29.7/35.6	I3	0.91	22.6/32.7
C4	1.13	27.2/36.8	C4	1.04	23.6/28.4
F18	0.94	31.4/33.0	S5	1.06	22.5/31.9
Y20	0.98	35.0/34.5	A16	1.08	27.2/31.9
D21	0.89	38.5/40.1	F18	0.84	25.6/32.7
N22	0.88	36.5/39.9	N19	0.85	28.6/39.5
T26	0.98	38.9/36.2	Y20	0.88	23.0/34.1
A31	0.95	33.8/39.3	D21	0.83	28.9/40.3
A46	0.88	27.9/47.3	N22	0.81	26.1/38.5
G77	1.12	24.9/45.5	I27	0.86	27.3/36.4
S82	0.82	32.2/46.1	K28	0.96	31.6/39.3
V86	0.96	23.4/36.5	L29	1.06	30.5/34.3
L147	1.00	33.6/30.3	I30	0.94	31.7/37.1
			Q111	0.81	32.2/35.7
			E145	0.81	21.5/31.8

I_{ox}/I_{red}: Relative peak intensity (height) between the oxidized (I_{ox}) and reduced (I_{red}) spectra.
Distance: The closest distance between the labeled sites of dU1 or dU2 and the indicated residue in the Fpr-HJ crystal structure (labeled as 1) and in RuvC-based docking model of Fpr-HJ complex (labeled as 2). The distances within 25 Å, which do not fit the PRE data, are highlighted in red.

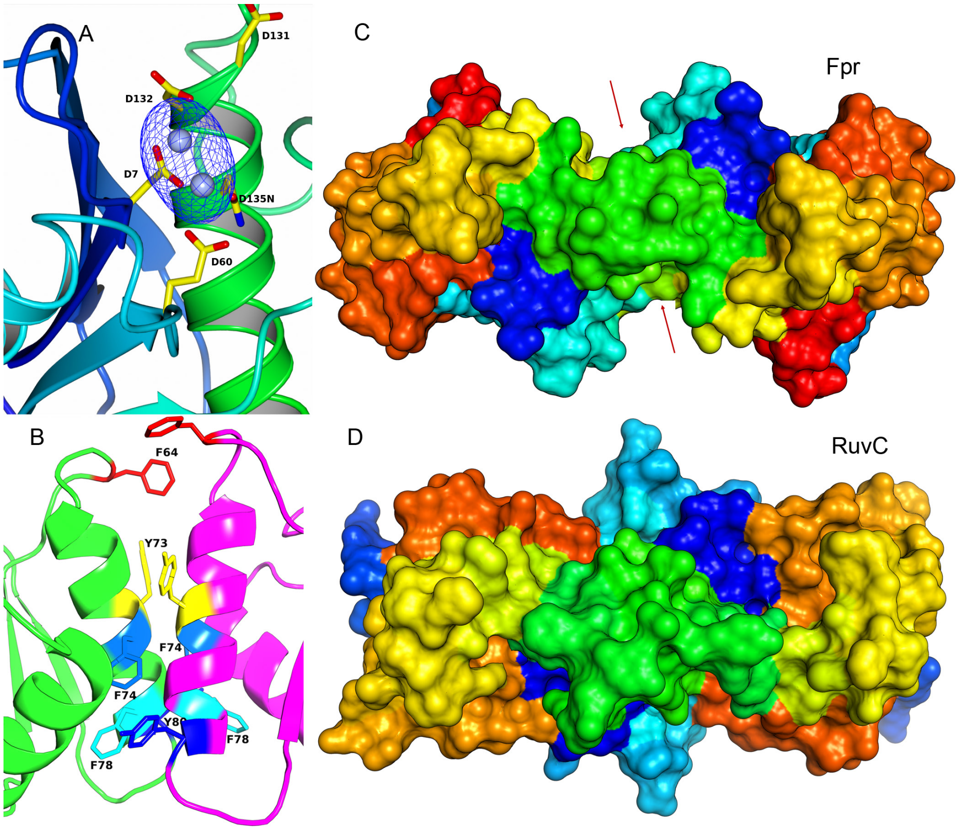

Supplementary Fig. S1. (A) Active site residues and the cadmium ions. The 2Fo-Fc map is shown in blue mesh contoured at 3.5σ level showing locations of the two cadmium ions. (B) The dimer interface of Fpr. Fpr forms homodimer by hydrophobic interactions involving F64, Y73, F74, F78 and Y80. (C, D) The overall structure of Fpr and *Tth*RuvC. The arrows indicate the basic side-grooves of Fpr. The protein chains are colored in a gradient of blue to red from the N- to the C-terminus.

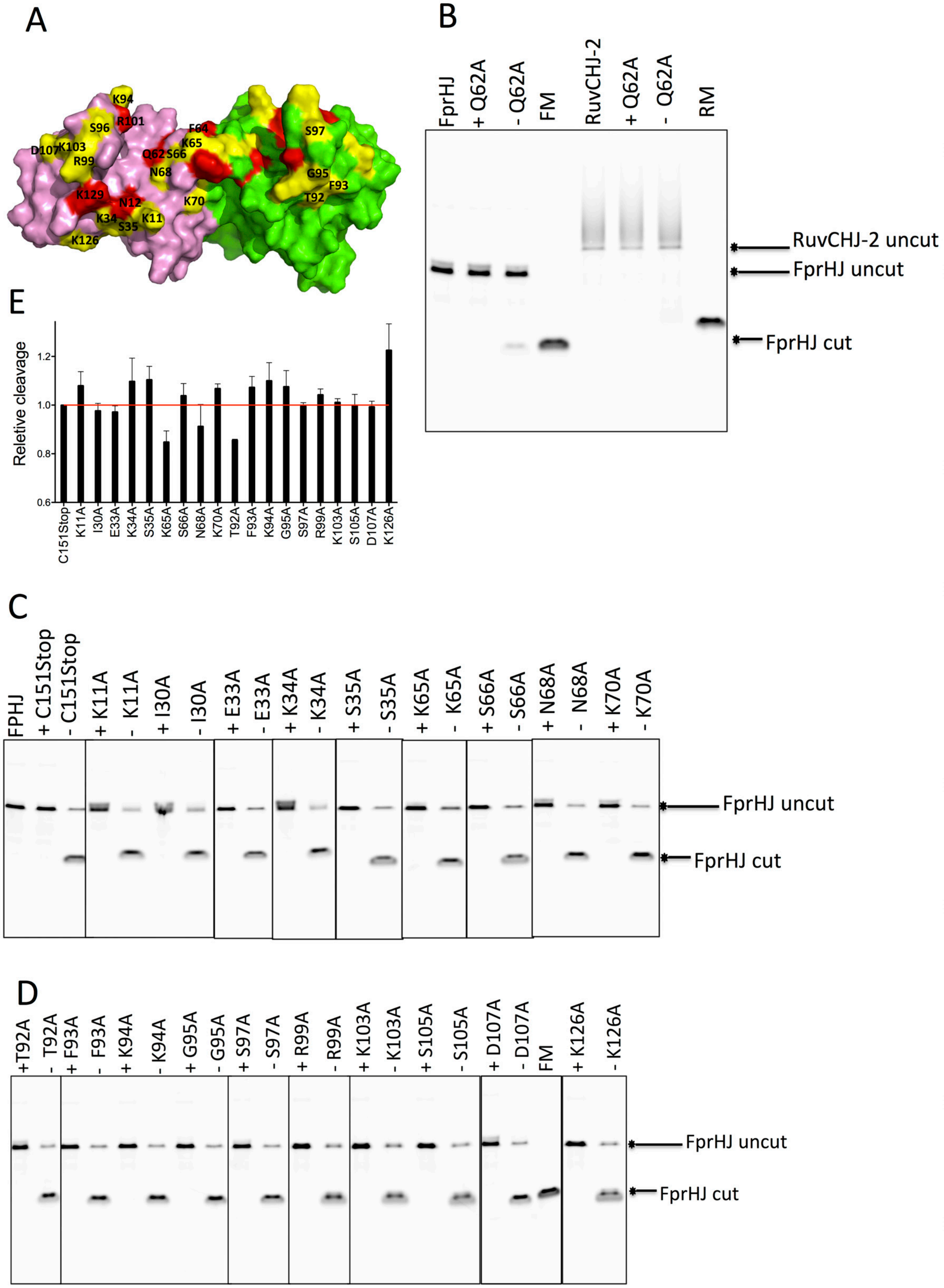
A
K94
R101
S96
D107
K103
R99
Q62
S66
K65
F64
N68
K70
K129
N12
K34
S35
K11
K126
S97
G95
F93
T92
B
FprHJ
+ Q62A
- Q62A
FM
RuvCHJ-2
+ Q62A
- Q62A
RM
RuvCHJ-2 uncut
FprHJ uncut
FprHJ cut
E
Reletive cleavage
C151Stop
K11A
I30A
E33A
K34A
S35A
K65A
S66A
N68A
K70A
T92A
F93A
K94A
G95A
S97A
R99A
K103A
S105A
D107A
K126A
C
FPHJ
+ C151Stop
- C151Stop
+ K11A
- K11A
+ I30A
- I30A
+ E33A
- E33A
+ K34A
- K34A
+ S35A
- S35A
+ K65A
- K65A
+ S66A
- S66A
+ N68A
- N68A
+ K70A
- K70A
FprHJ uncut
FprHJ cut
D
+ T92A
- T92A
+ F93A
- F93A
+ K94A
- K94A
+ G95A
- G95A
+ S97A
- S97A
+ R99A
- R99A
+ K103A
- K103A
+ S105A
- S105A
+ D107A
- D107A
FM
+ K126A
- K126A
FprHJ uncut
FprHJ cut

Supplementary Fig. S2. Comparison of the HJ cleavage activities of Fpr C151stop with surface mutants. (A) The top view of surface residues in apo Fpr, with the two monomers shown in pink and green. Surface residues are highlighted in yellow or red. (B) Cleavage of FprHJ and RuvCHJ-2 by C151Stop Q62A. The reaction mixture (20μl) containing Fpr dimer (300nM) with FprHJ (100 nM) or RuvCHJ-2 (100 nM) was incubated at 37°C for 10 min. The reaction products were analyzed on a denaturing gel. FprHJ, FprHJ only in reaction buffer as a control. FM, FprHJ marker. RuvCHJ-2, RuvCHJ-2 only in reaction buffer as a control. RM, RuvCHJ marker. (C, D) Cleavage of FprHJ by Fpr with the indicated mutations of surface residues or those near the active sites. The reaction system was the same as above and the reaction products were analyzed on denaturing gels. (E) Quantification of the cleavage products of above mutants relative to that of Fpr C151Stop. The red solid line shows the cleavage ability of Fpr C151stop. The lanes labeled "+" were with EDTA to inhibit the reaction, as negative controls. Borders of cropped gels are highlighted by black lines. Each DNA cleavage experiment was repeated 3 times and a representative cropped gel is shown in the figure. Original full-length gels for Supplementary Fig. S2 are shown in Supplementary Fig. S10.

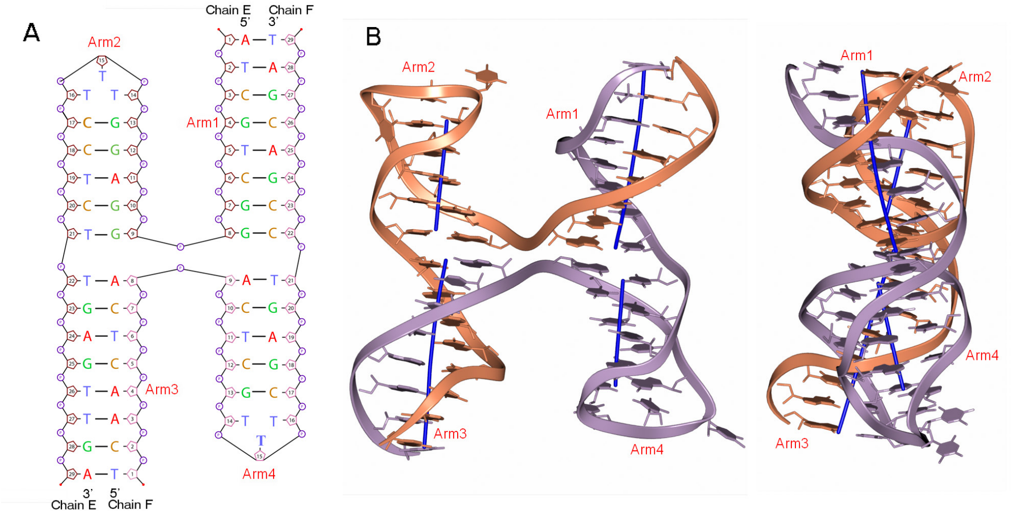

Supplementary Fig. S3. HJ8/5 DNA used in crystallization and its conformation in the Fpr-HJ8/5 complex crystal. (A) Schematic diagram of the DNA molecule with base-pairs indicated. (B) H-stacked conformation of the DNA molecule in the Fpr-HJ8/5 complex. The helical axis of each double-stranded DNA arm is shown by a blue line.

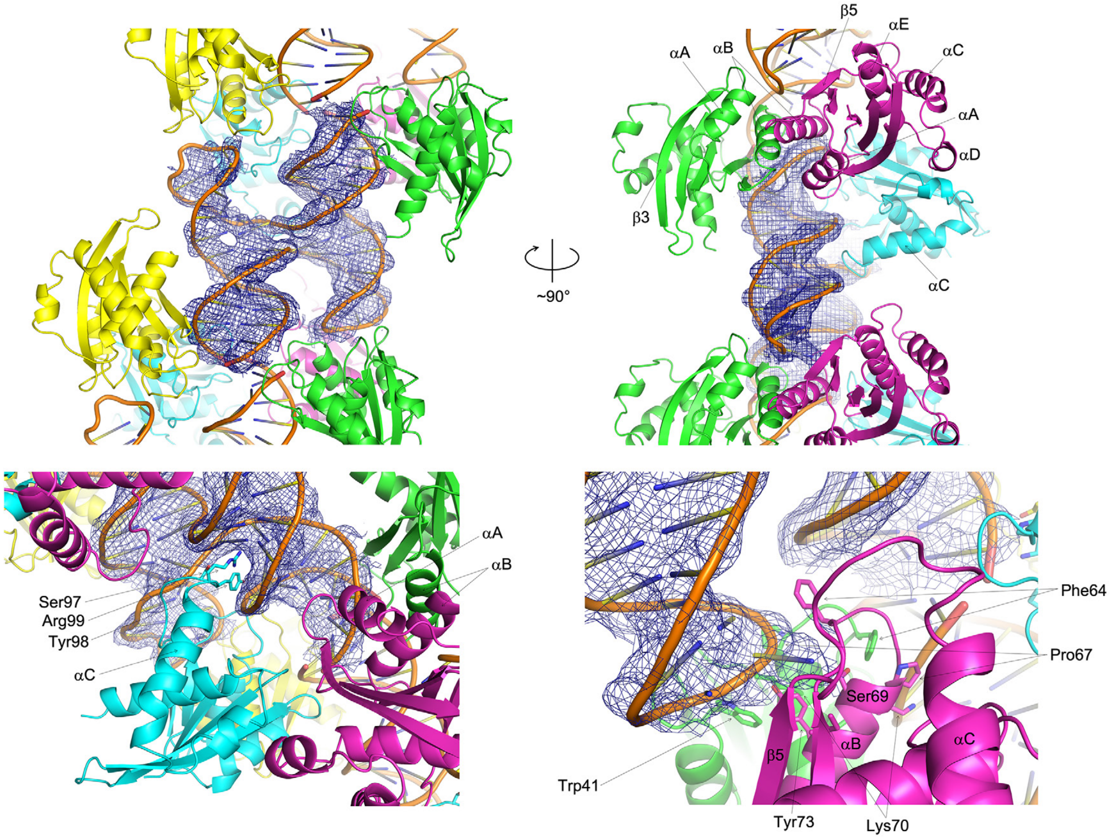

Supplementary Fig. S4. Fpr-HJ8/5 complex crystal structure. The 2mFo-DFc electron density map at 1.0σ contour level is shown around a HJ DNA molecule (3.0 Å cutoff). Four crystallographically independent Fpr molecules are colored differently, and some of the side-chains making DNA interactions or secondary structure elements are labeled.

A

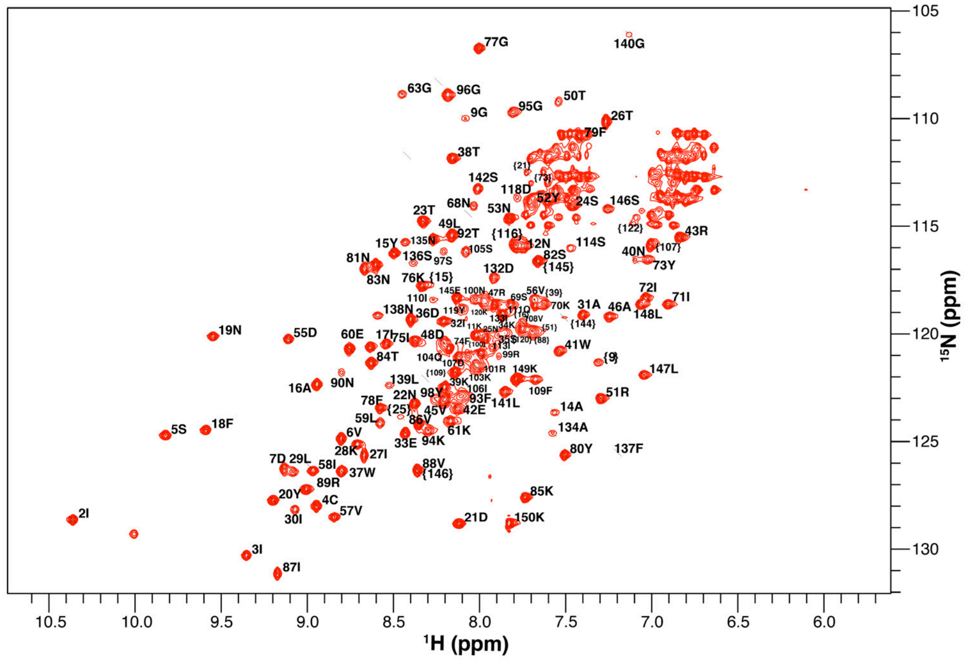

B

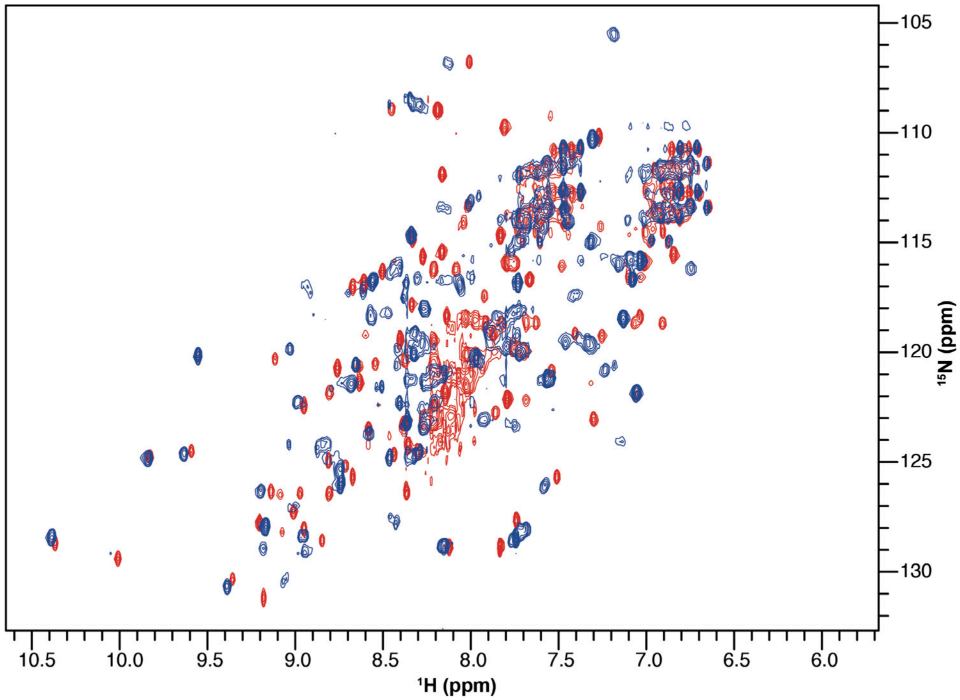

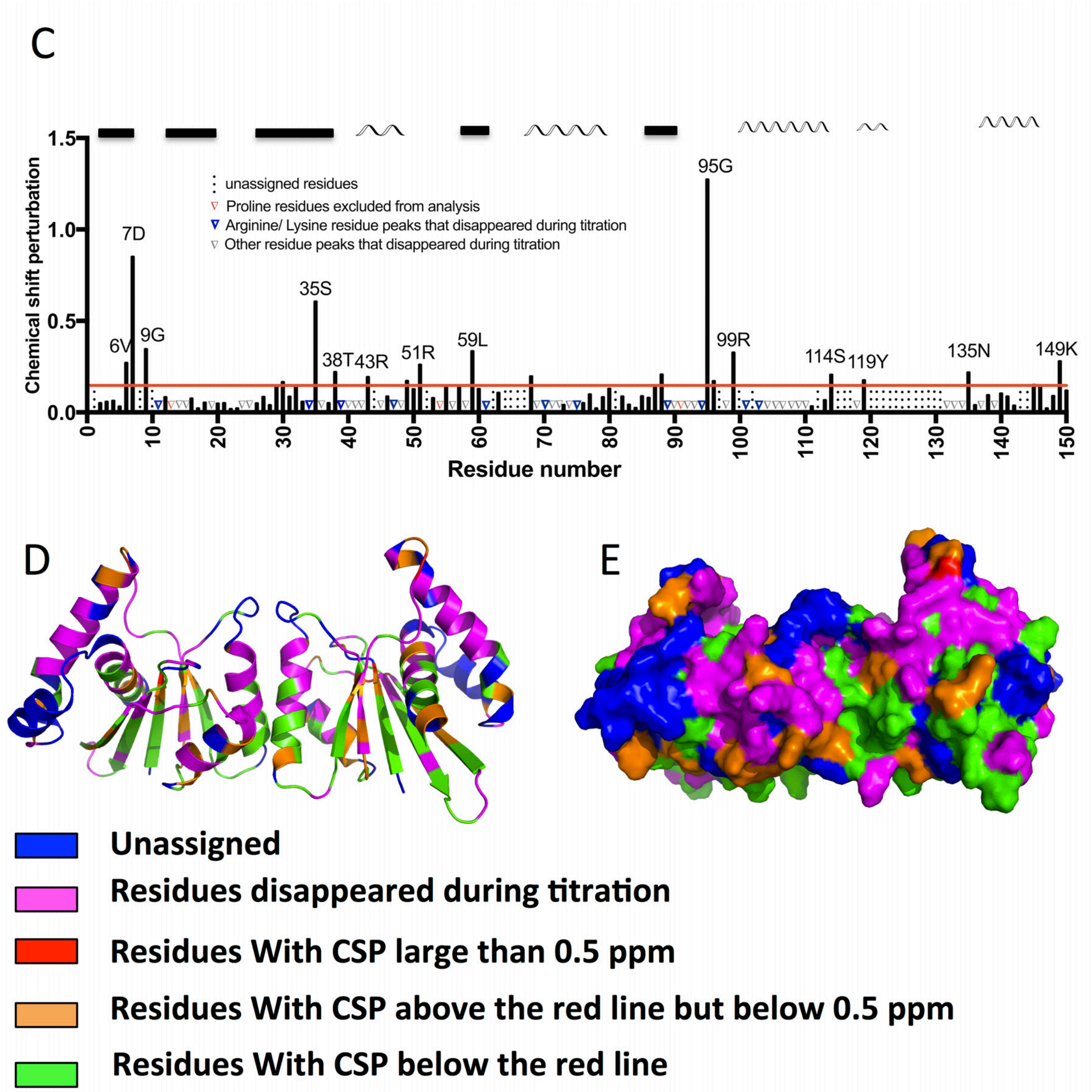

Supplementary Fig. S5. NMR analyses of the interaction between Fpr C151Stop D135N and HJ8/5 in solution. (A) 2D $^{1}H,^{15}N$ HSQC spectrum and backbone resonance assignments of Fpr C151Stop D135N. Residues from K120 to D131 were not assigned. (B) Spectral overlay of Fpr C151Stop D135N only (red) and C151Stop D135N dimer with HJ8/5 (blue) at the ratio of 1:3. (C) Per residue chemical shift perturbations observed in titration of HJ DNA into Fpr dimer. The solid red line crosses the graph at $1\sigma_{0\ \mathrm{corr}}$ (corrected standard deviation (4,5)). Unassigned residues are marked by black dotted lines on the graph. The red triangles on the graph represent three proline residues that do not have the NH group. Blue and grey triangles indicate residues whose resonances disappeared due to significant

broadening during the titration. (D) Ribbon model and (E) Surface representation of Fpr dimer with residues colored according to the magnitude of CSPs. Residues with significant changes are highlighted by red color on the structure, those with moderate changes in orange, and smaller changes in green. Residues whose backbone resonances disappeared during titration are in magenta, and unassigned residues are in blue.

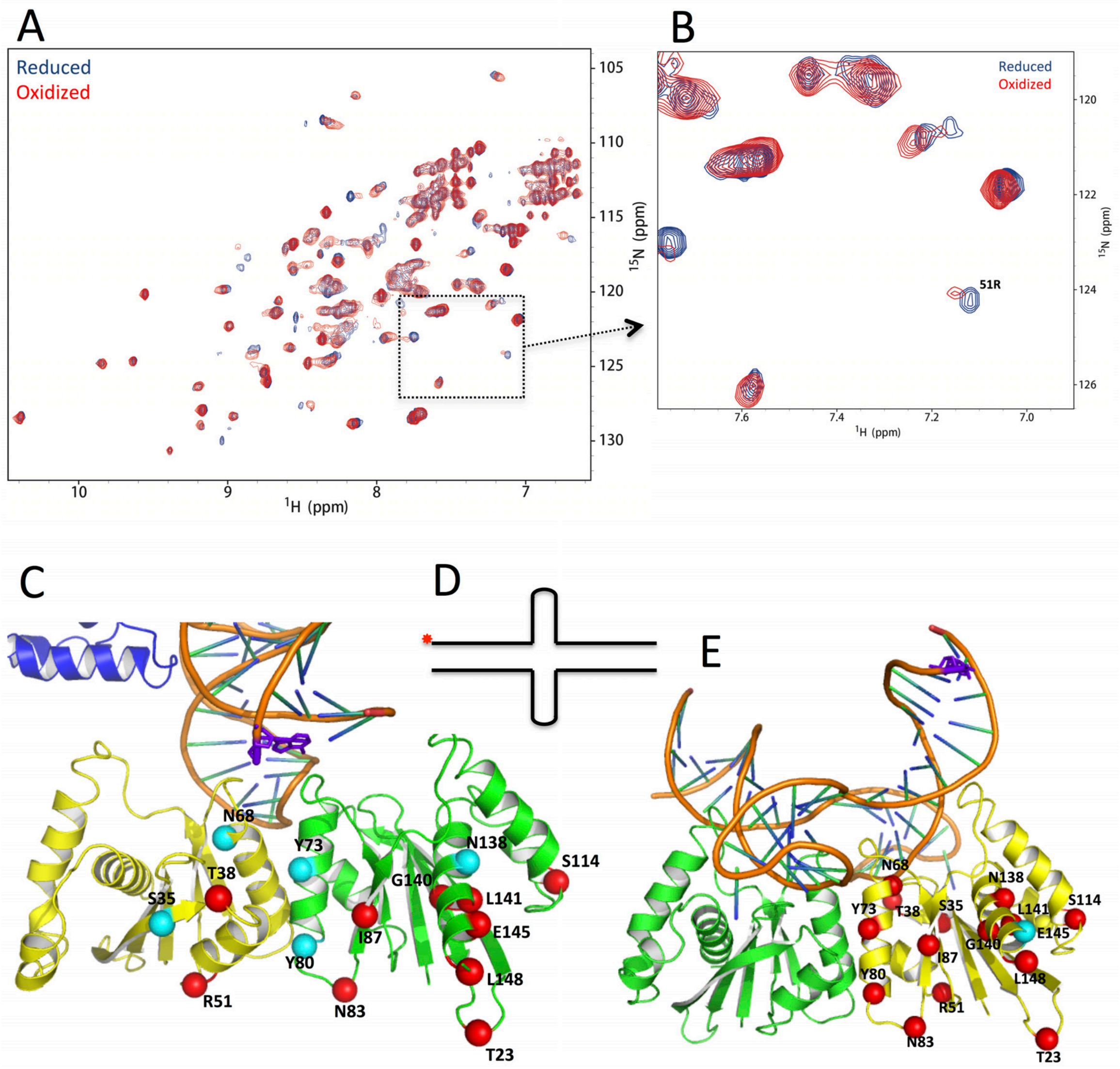

Supplementary Fig. S6. Interaction between HJ8/5 with Du1 spin-label and Fpr C151Stop D135N in solution. (A, B) Spectral overlay of Fpr with spin-labeled dU1 HJ8/5 in oxidized (red) and reduced states (blue). (C) Mapping of the residues affected by PRE (I_{ox}/I_{red} < 0.8) with dU1 HJ8/5 on the crystal structure of Fpr-DNA complex. The spin-labeled position in DNA is indicated in purple. For each of the PRE-affected residues, the closer position from the spin-label is shown. The residues in cyan are within 25 Å from the spin-label, whereas those in red are farther than 25 Å. (D) A schematic diagram of deoxy-4-thiouridine (red dot)-containing dU1 HJ8/5. (E) Mapping of the residues affected by PRE (I_{ox}/I_{red} < 0.8) with dU1 HJ8/5 on the "canonical" Fpr-HJ8/5 complex model based on the RuvC-HJ complex crystal structure. The spin-labeled position in DNA is indicated in purple. For each of the PRE-affected residues, the closer position from the spin-label within the Fpr dimer is shown. The color scheme is same as that in (C).

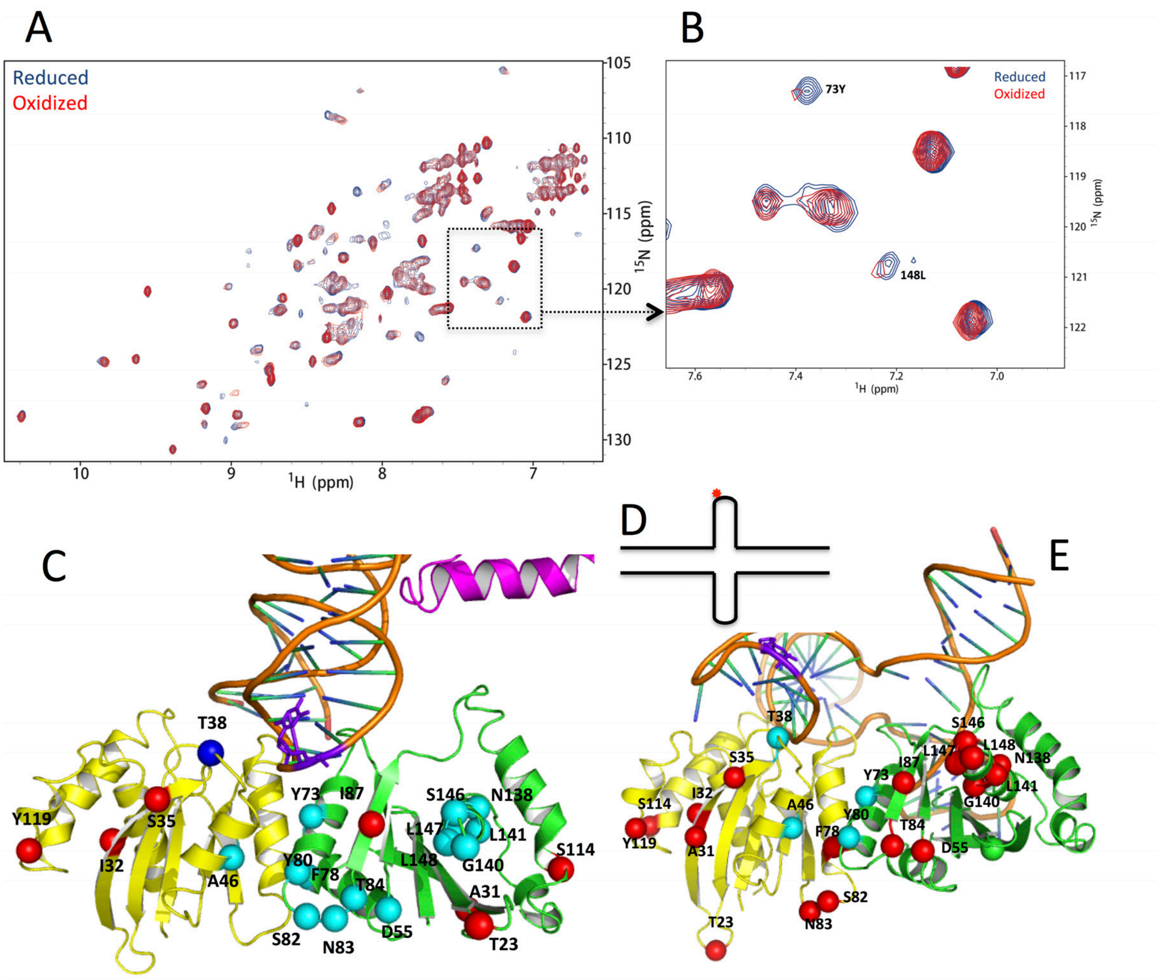

Supplementary Fig. S7. Interaction between HJ8/5 with Du2 spin-label and Fpr C151Stop D135N in solution. (A, B) Spectral overlay of Fpr with spin-labeled dU2 HJ8/5 in oxidized (red) and reduced state (blue). (C) Mapping of the residues affected by PRE ($I_{ox}/I_{red} < 0.8$) with dU2 HJ8/5 on the crystal structure of Fpr-DNA complex. The spin-labeled position in DNA is indicated in purple. For each of the PRE-affected residues, the closer position from the spin-label is shown. The residues in cyan are within 25 Å from the spin-label, whereas those in red are farther than 25 Å. T38 in blue is less than 12 Å from the spin-label. (D) A schematic diagram of deoxy-4-thiouridine (red dot)-containing dU2 HJ8/5. (E) Mapping of the residues affected by PRE ($I_{ox}/I_{red} < 0.8$) with dU2 HJ8/5 on the “canonical” Fpr-HJ8/5 complex model based on the RuvC-HJ complex crystal structure. The spin-labeled position in DNA is indicated in purple. For each of the PRE-affected residues, the closer position from the spin-label within the Fpr dimer is shown. The color scheme is same as that in (C).

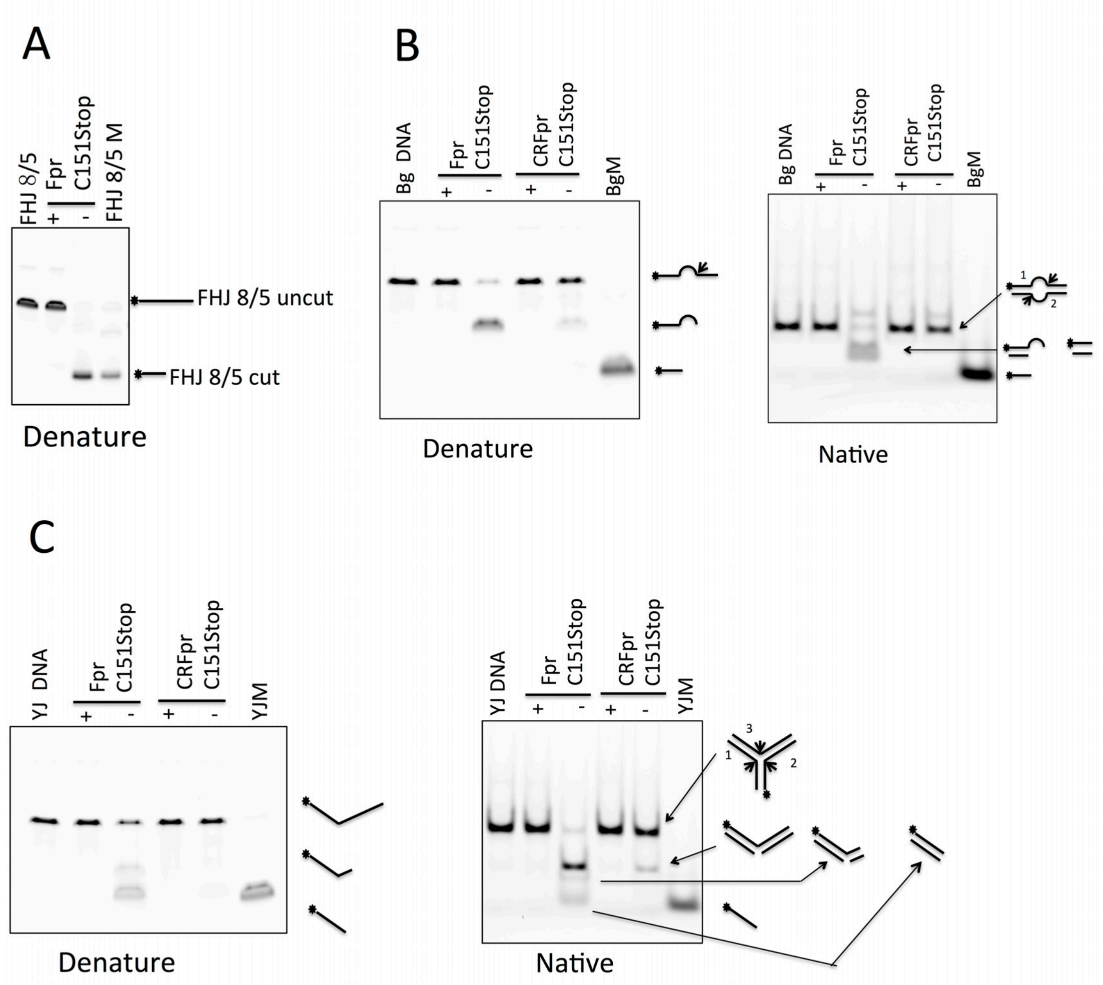

Supplementary Fig. S8. Comparison of the Bulged and Y junction DNA cleavage activities of Fpr C151stop and CRFpr C151 stop. (A) Cleavage of Florescently labeled FHJ8/5 substrate by Fpr. The reaction mixture (20µl) containing Fpr dimer (300nM) with FHJ8/5 (100 nM) was incubated at 37°C for 10 min. The reaction products were analyzed on denaturing gel. FHJ8/5, FHJ8/5 only in reaction buffer as a control. FHJ8/5M, FHJ8/5 marker. (B) Cleavage of Bulged substrate by Fpr and CRFpr. The reaction mixture (20µl) containing Fpr dimer (600nM) or CrFpr dimer (600nM) and Bulge DNA (100 nM) was incubated at 37°C for 1 hour. The reaction products were separately analyzed on denaturing (left) and native gels (right). Bg DNA, Bugle DNA only in reaction buffer as a control. BgM, Bulge marker. (C) Cleavage of Y Junction DNA by Fpr and CRFpr. The reaction system was the same as that described above for Bulge DNA. YJ DNA, Y junction only in reaction buffer as a control. YJM, Y junction Marker. The lanes labeled "+" were with EDTA to inhibit the reaction, as negative controls. Each DNA cleavage experiment was repeated 3 times and a representative gel is shown in the figure. Original full-length gels for Supplementary Fig. S8 are shown in Supplementary Fig. S11.

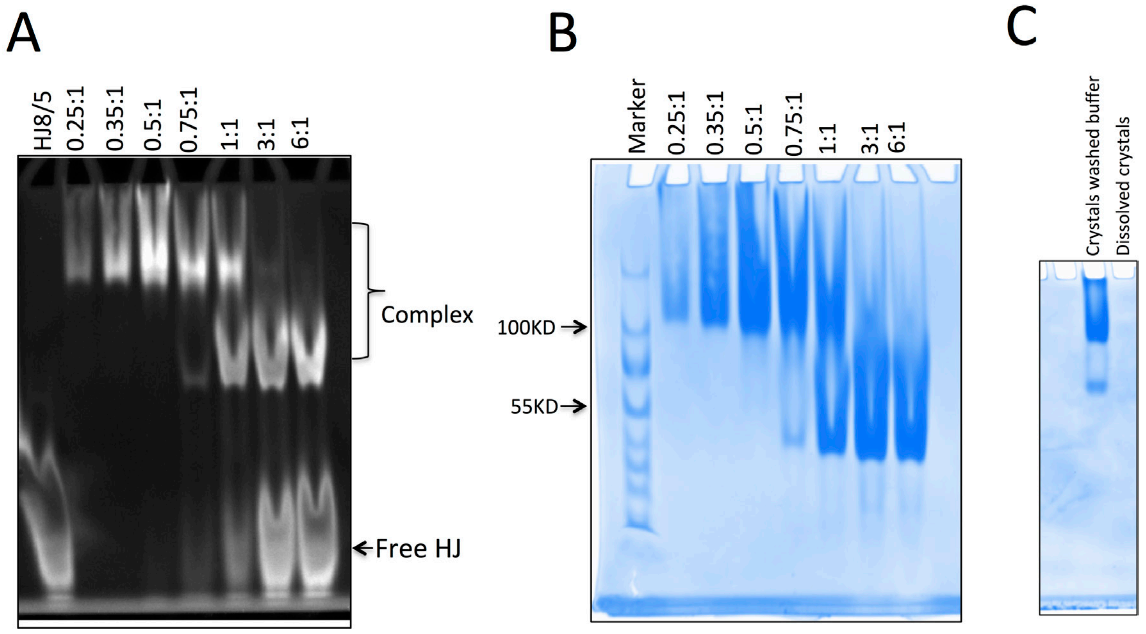

Supplementary Fig. S9. Original full-length gels for Figure 4.

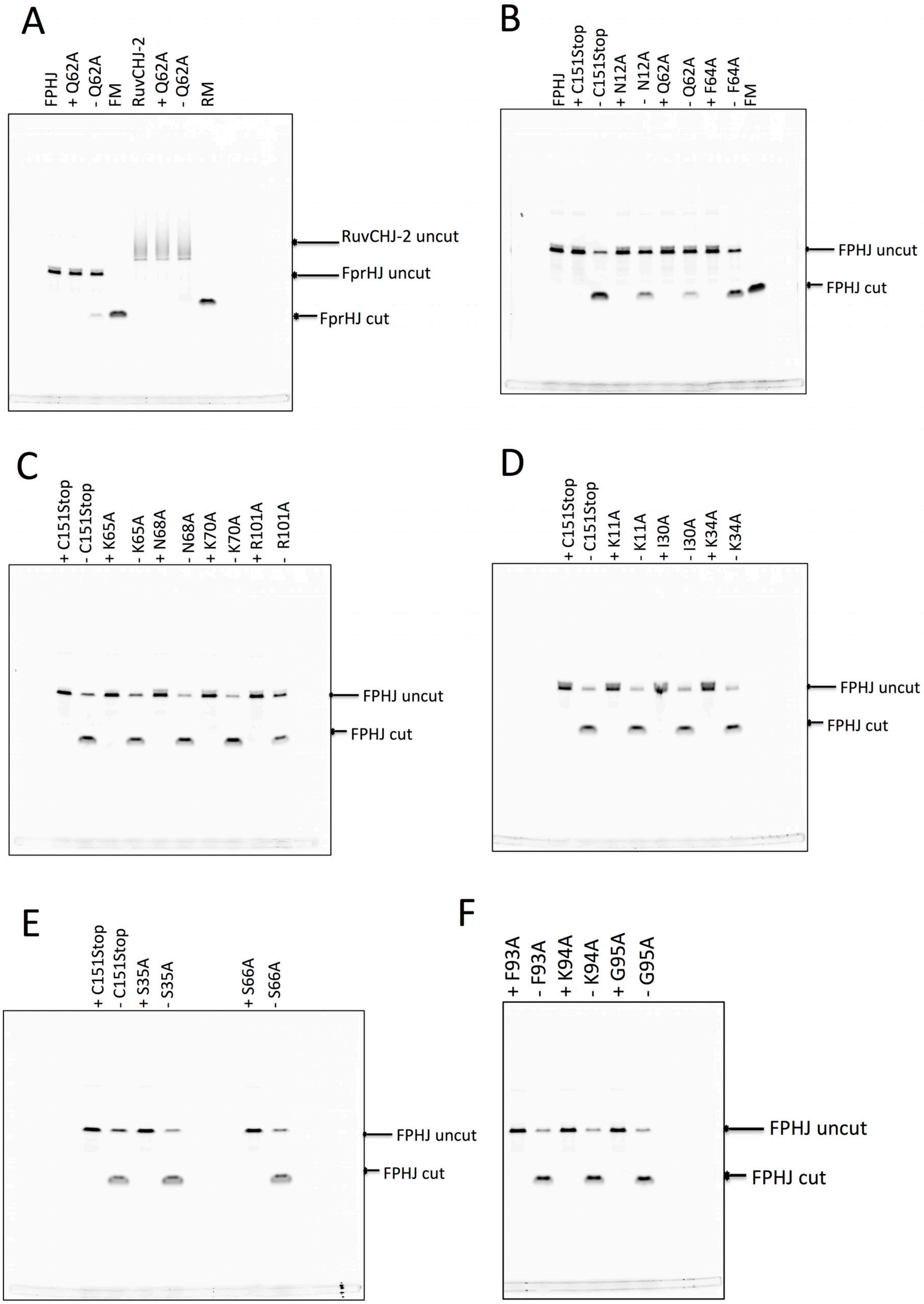
A
FPHJ
+ Q62A
- Q62A
FM
RuvCHJ-2
+ Q62A
- Q62A
RM
RuvCHJ-2 uncut
FprHJ uncut
FprHJ cut
B
FPHJ
+ C151Stop
- C151Stop
+ N12A
- N12A
+ Q62A
- Q62A
+ F64A
- F64A
FM
FPHJ uncut
FPHJ cut
C
+ C151Stop
- C151Stop
+ K65A
- K65A
+ N68A
- N68A
+ K70A
- K70A
+ R101A
- R101A
FPHJ uncut
FPHJ cut
D
+ C151Stop
- C151Stop
+ K11A
- K11A
+ I30A
- I30A
+ K34A
- K34A
FPHJ uncut
FPHJ cut
E
+ C151Stop
- C151Stop
+ S35A
- S35A
+ S66A
- S66A
FPHJ uncut
FPHJ cut
F
+ F93A
- F93A
+ K94A
- K94A
+ G95A
- G95A
FPHJ uncut
FPHJ cut

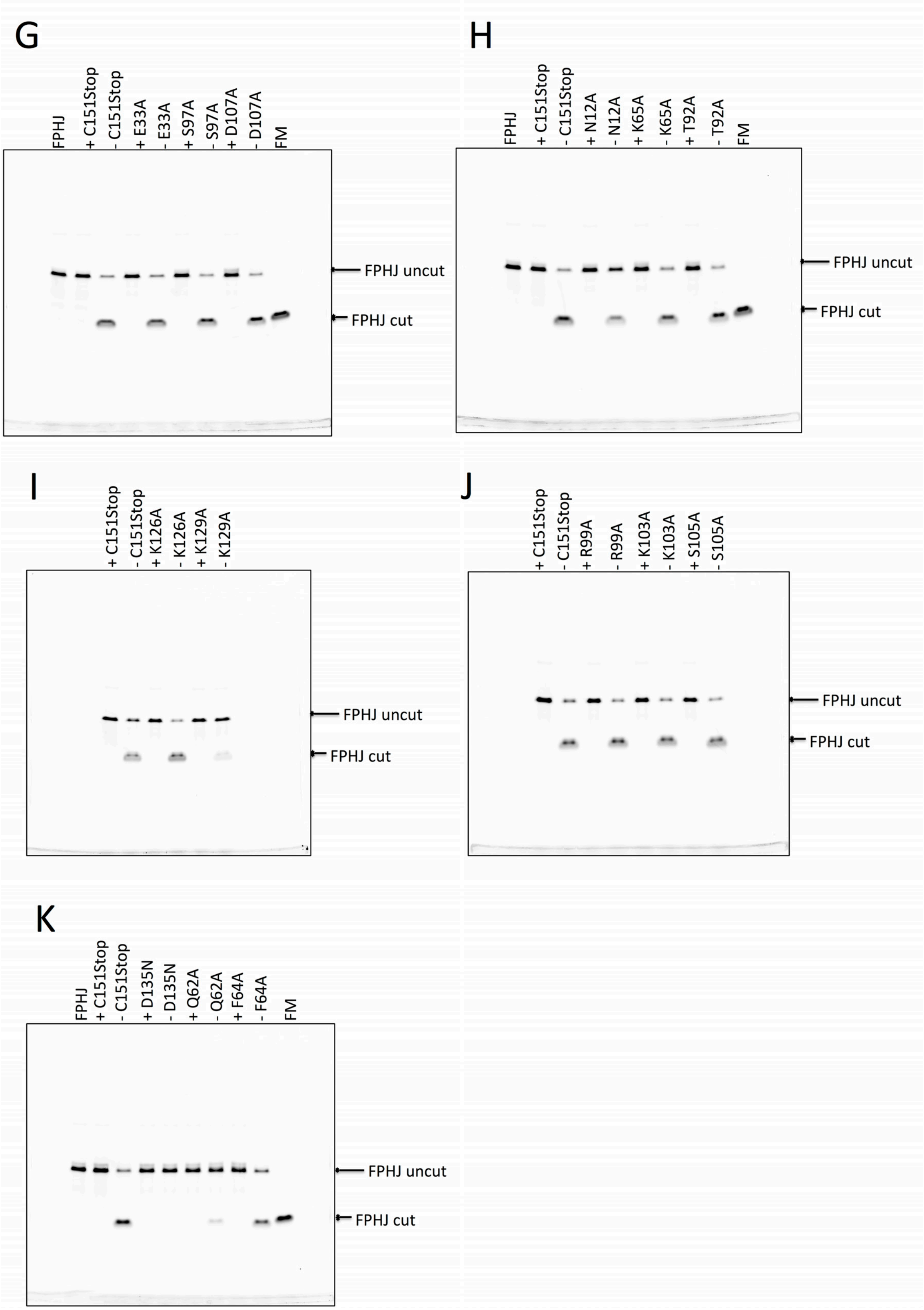
G
FPHJ
+ C151Stop
- C151Stop
+ E33A
- E33A
+ S97A
- S97A
+ D107A
- D107A
FM
FPHJ uncut
FPHJ cut
H
FPHJ
+ C151Stop
- C151Stop
+ N12A
- N12A
+ K65A
- K65A
+ T92A
- T92A
FM
FPHJ uncut
FPHJ cut
I
+ C151Stop
- C151Stop
+ K126A
- K126A
+ K129A
- K129A
FPHJ uncut
FPHJ cut
J
+ C151Stop
- C151Stop
+ R99A
- R99A
+ K103A
- K103A
+ S105A
- S105A
FPHJ uncut
FPHJ cut
K
FPHJ
+ C151Stop
- C151Stop
+ D135N
- D135N
+ Q62A
- Q62A
+ F64A
- F64A
FM
FPHJ uncut
FPHJ cut

Supplementary Fig. S10. Original full-length gels for Figure 2A and Supplementary Fig. S2 B, C and D. (A) Original full-length gels for Supplementary Fig. S2 B. (B) Original full-length gel for cleavage of FprHJ DNA by C151Stop, N12A, Q62A and F64A in Figure 2A. (C) Original full-length gel for cleavage of FprHJ DNA by K65A, N68A, K70A in Supplementary Fig. S2 C and R101A in Figure 2A. (D) Original full-length gel for cleavage of FprHJ DNA by K11A, I30A and K34A in Supplementary Fig. S2 C. (E) Original full-length gel for cleavage of FprHJ DNA by S35A and S66A in Supplementary Fig. S2 C. (F) Original full-length gel for cleavage of FprHJ DNA by F93A, K94A and G95A in Supplementary Fig. S2 D. (G) Original full-length gel for cleavage of FprHJ DNA by E33A in Supplementary Fig. S2 C, S97A and D107A in Supplementary Fig. S2 D. (H) Original full-length gel for cleavage of FprHJ DNA by T92A in Supplementary Fig. S2 D. (I) Original full-length gel for cleavage of FprHJ DNA by K126A and K129A in Supplementary Fig. S2 D. (J) Original full-length gel for cleavage of FprHJ DNA by R99A, K103A and S105A in Supplementary Fig. S2 D. (K) Original full-length gel for cleavage of FprHJ DNA by D135N in Figure 2A. Each DNA cleavage experiment was repeated 3 times and a representative gel is shown in the figure.

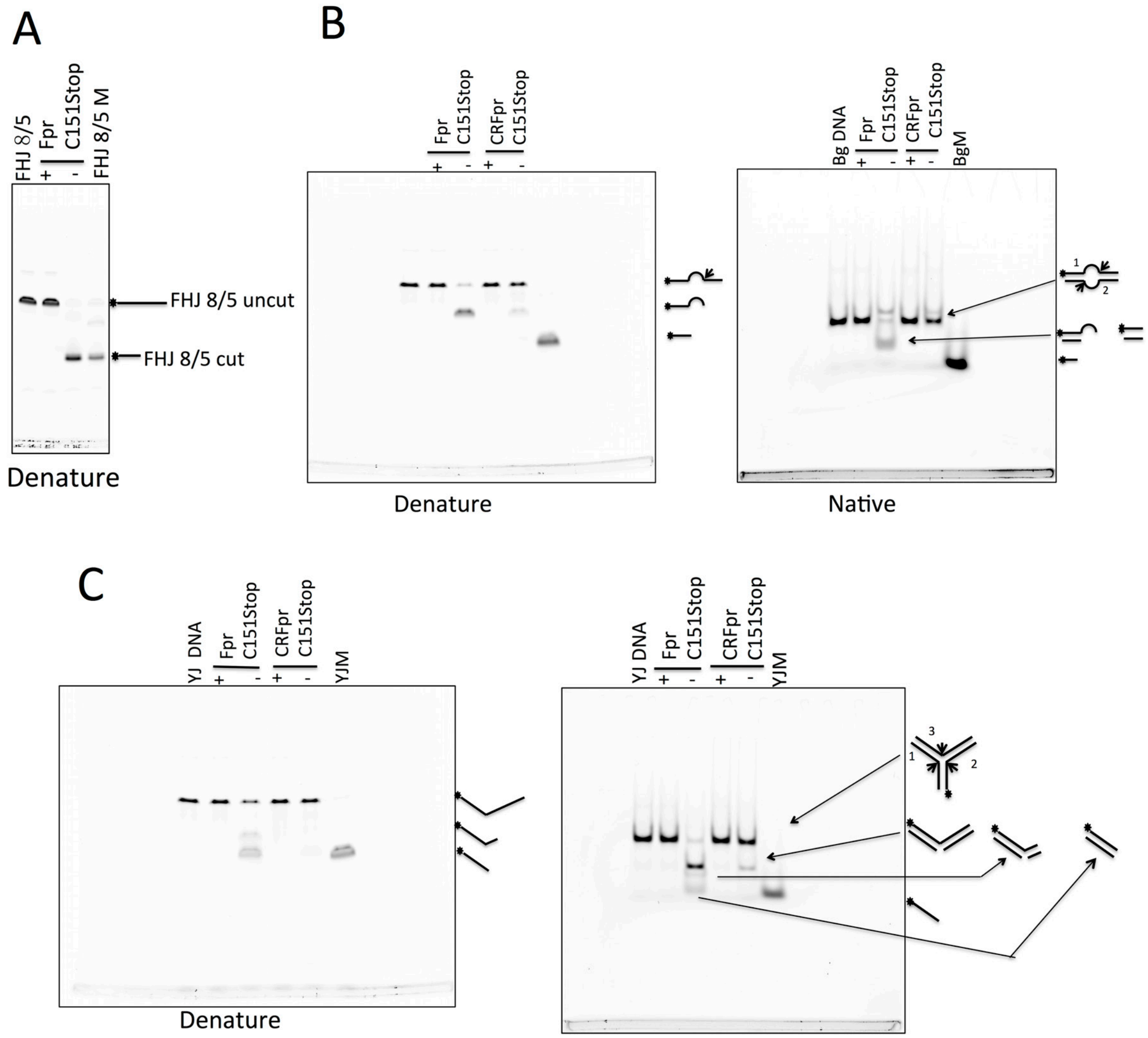

Supplementary Fig. S11. Original full-length gels for Supplementary Fig. S8. Each DNA cleavage experiment was repeated 3 times and a representative gel is shown in the figure.

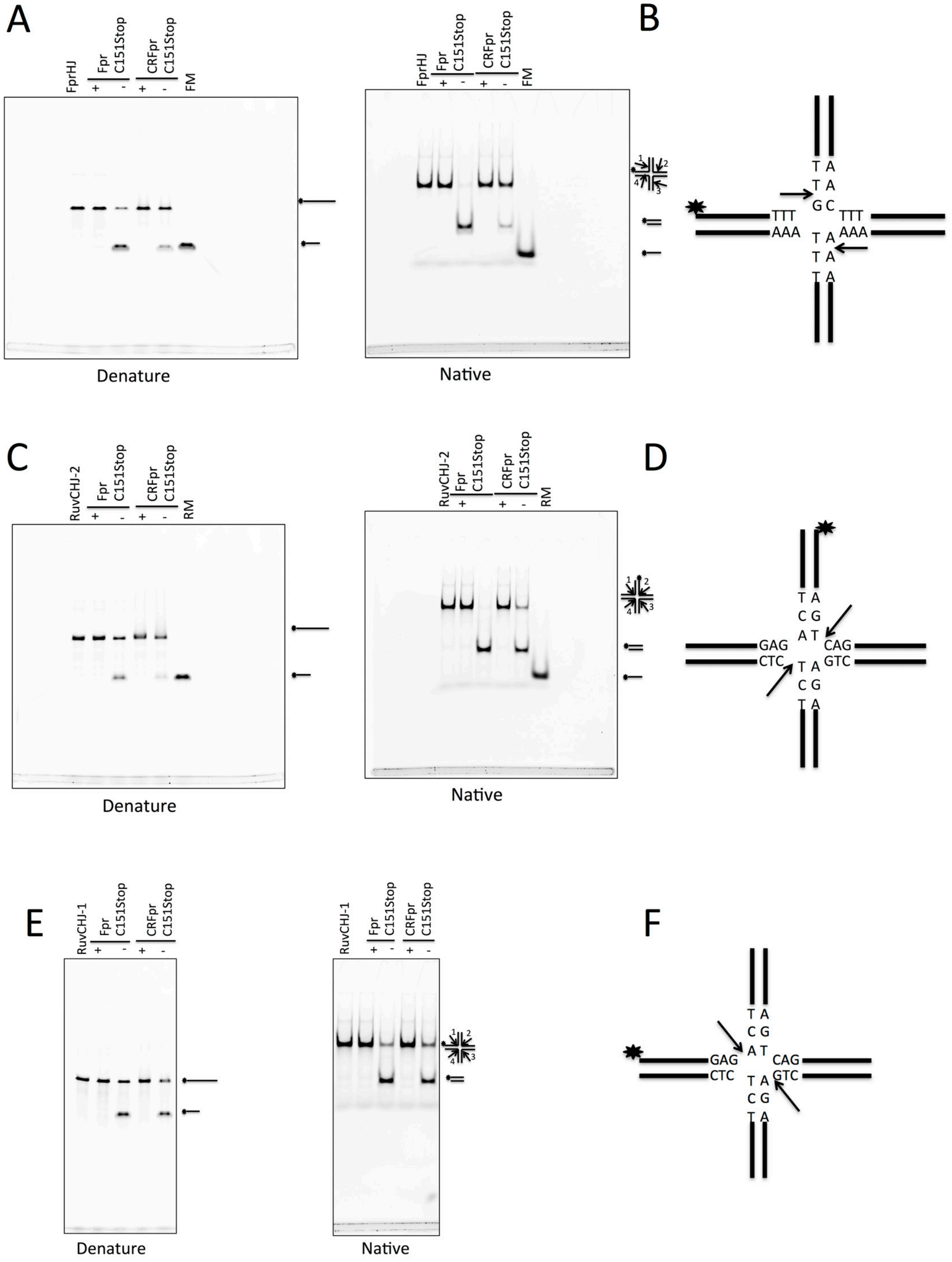

Supplementary Fig. S12. Original full-length gels for Figure 6. Each DNA cleavage experiment was repeated 3 times and a representative gel is shown in the figure.

Reference:

1. Hadden, J.M., Declais, A.-C., Carr, S.B., Lilley, D.M.J. and Phillips, S.E.V. (2007) The structural basis of Holliday junction resolution by T7 endonuclease. *Nature*, **449**, 621-U615.
2. Culyba, M.J., Hwang, Y., Minkah, N. and Bushman, F.D. (2009) DNA Binding and Cleavage by the Fowlpox Virus Resolvase. *Journal of Biological Chemistry*, **284**, 1190-1201.
3. Chen, L., Shi, K., Yin, Z. and Aihara, H. (2013) Structural asymmetry in the Thermus thermophilus RuvC dimer suggests a basis for sequential strand cleavages during Holliday junction resolution. *Nucleic Acids Research*, **41**, 648-656.
4. Schumann, F.H., Riepl, H., Maurer, T., Gronwald, W., Neidig, K.P. and Kalbitzer, H.R. (2007) Combined chemical shift changes and amino acid specific chemical shift mapping of protein-protein interactions. *Journal of Biomolecular Nmr*, **39**, 275-289.
5. Williamson, M.P. (2014) Using chemical shift perturbation to characterise ligand binding (vol 73, pg 1, 2013). *Progress in Nuclear Magnetic Resonance Spectroscopy*, **80**, 64-64.
